# Distributed probing of chromatin structure *in vivo *reveals pervasive chromatin accessibility for expressed and non-expressed genes during tissue differentiation in *C. elegans*

**DOI:** 10.1186/1471-2164-11-465

**Published:** 2010-08-06

**Authors:** Ky Sha, Sam G Gu, Luiz C Pantalena-Filho, Amy Goh, Jamie Fleenor, Daniel Blanchard, Chaya Krishna, Andrew Fire

**Affiliations:** 1 Depts. of Pathology and Genetics, Stanford University School of Medicine, 300 Pasteur Drive, Palo Alto CA, USA; 2 Carnegie Institution of Washington, 115 West University Parkway, Baltimore MD, USA; 3 Biology Department, Johns Hopkins University, 3400 North Charles St., Baltimore MD, USA

## Abstract

**Background:**

Tissue differentiation is accompanied by genome-wide changes in the underlying chromatin structure and dynamics, or epigenome. By controlling when, where, and what regulatory factors have access to the underlying genomic DNA, the epigenome influences the cell's transcriptome and ultimately its function. Existing genomic methods for analyzing cell-type-specific changes in chromatin generally involve two elements: (i) a source for purified cells (or nuclei) of distinct types, and (ii) a specific treatment that partitions or degrades chromatin by activity or structural features. For many cell types of great interest, such assays are limited by our inability to isolate the relevant cell populations in an organism or complex tissue containing an intertwined mixture of other cells. This limitation has confined available knowledge of chromatin dynamics to a narrow range of biological systems (cell types that can be sorted/separated/dissected in large numbers and tissue culture models) or to amalgamations of diverse cell types (tissue chunks, whole organisms).

**Results:**

Transgene-driven expression of DNA/chromatin modifying enzymes provides one opportunity to query chromatin structures in expression-defined cell subsets. In this work we combine *in vivo *expression of a bacterial DNA adenine methyltransferase (DAM) with high throughput sequencing to sample tissue-specific chromatin accessibility on a genome-wide scale. We have applied the method (DALEC: Direct Asymmetric Ligation End Capture) towards mapping a cell-type-specific view of genome accessibility as a function of differentiated state. Taking advantage of *C. elegans *strains expressing the DAM enzyme in diverse tissues (body wall muscle, gut, and hypodermis), our efforts yield a genome-wide dataset measuring chromatin accessibility at each of 538,000 DAM target sites in the *C. elegans *(diploid) genome.

**Conclusions:**

Validating the DALEC mapping results, we observe a strong association between observed coverage by nucleosomes and low DAM accessibility. Strikingly, we observed no extended regions of inaccessible chromatin for any of the tissues examined. These results are consistent with "local choreography" models in which differential gene expression is driven by intricate local rearrangements of chromatin structure rather than gross impenetrability of large chromosomal regions.

## Background

Recent advances in sequencing technology have allowed experimentalists a global view of the relationship between chromatin structure and genomic activity during development. By combining chromatin immunoprecipitation (ChIP) with high throughput sequencing or DNA microarrays (ChIP-Seq or ChIP-chip), it is possible to query the genomic localizations of specific transcription factors, histone modifications, and chromatin remodelling factors. Chromatin state maps from ChIP-Seq and ChIP-chip experiments along with data from genome-wide nuclease accessibility studies can be used to define molecular landscapes (including transcription start sites, regions of active transcription, enhancers, euchromatin, heterochromatin, etc.) on a genome-wide scale. Implicit in this analysis is the assumption that a cell's chromatin signature, or its epigenome, will be highly diagnostic of function.

One metric of chromatin structure is accessibility of the DNA in bulk chromatin. DNA-modifying enzymes such as nucleases [1,2] and methyltransferases [3-5] have proven to be useful tools for defining susceptible regions. Accessible DNA may in some cases define regions of "open chromatin" that allow access to DNA binding factors such as transcription factors. In contrast, less accessible DNA may define regions of relatively compact chromatin and is often characterized by transcriptional inactivity.

Cleavage of chromatin by micrococcal nuclease (MNase) has been a standard method for examining nucleosome positioning and regional accessibility, both *in vitro *and *in situ *[6,7]. Despite the substantial information that can come from MNase studies, this enzyme is known to have specific sequence and structural preferences [8-10], generating a well-recognized need for additional reagents and methods to independently survey genome accessibility. Several alternative nuclease or other approaches have been used for localized studies of accessibility [2,11-15], each has its own potential advantages and potential biases.

One limitation of available methods for genomewide analysis of chromatin structures in specific cell types has been the need to isolate the individual cell type of interest in considerable bulk. Epigenome characterization methods using nucleases and other destructive probes have thus been applied only in the narrow range of biological systems where individual cell types can be isolated (or in whole organisms or mixed-cell-type tissues, where the results represent an amalgamation of the numerous constituent cell types). For many of the most interesting biological questions, the cell groups of interest are surrounded by (and embedded in) other very different cell types, making uniform-cell-type preparations impossible on the scale currently needed for genome-wide analysis. As one means of addressing this challenge, transgene-driven expression can be used to produce a specific probe in a defined cell type. This type of approach requires a probe that can detectably modify chromatin or DNA without killing or substantially disturbing the relevant cells.

DNA adenine methyltransferase (DAM) catalyzes the addition of a methyl group to the adenine base in the sequence GATC [16]. DAM activities are used by a number of bacterial phages and prokaryotes. In *E. coli*, DAM is involved in a multitude of cellular processes, including DNA replication, mismatch repair, control of gene expression, and restriction-modification immunity [17,18].

DAM from *E. coli *has been used to analyze chromatin structure in eukaryotic cells [19-21] and there is good localized agreement between chromatin structure inferred using DAM and other techniques, such as nuclease hypersensitivity mapping [4,22]. Importantly, sensitivity to DAM methylation correlates well with transcriptional activity [23-25]. These results support the use of susceptibility to DAM methylation as a measure of chromatin structure. Key advantages of using DAM to probe eukaryotic chromatin structure are (i) the ability to assay accessibility in a living cell, (ii) the ability to assay designated sets of cells or tissues in complex biological samples using transgenes with regulated promoters, and (iii) the lack of any known background of DAM activity in eukaryotes. The addition of 6-methyl adenosines at sites in eukaryotic genome is apparently well tolerated in a variety of tissues, as functional expression of DAM methyltransferase in yeast, Drosophila, and mammalian cells has not resulted in any apparent phenotypes [5,19,22,26,27].

Previous studies of chromatin structure using DAM were limited to the investigation of only a few loci due to the low throughput nature of Southern blotting experiments. In this article, we describe a method that couples DAM methylation to high throughput sequencing. We termed our method DALEC and applied it to investigate *in vivo *chromatin structure in three transgenic *C. elegans *strains, each expressing DAM from a tissue-specific promoter. We provide evidence that genic regions remain in an accessible state that can be probed with DAM activity even when not expressed, with features of chromatin structure inferred from DAM accessibility concordant with nucleosome positioning and expression data derived from independent sources.

## Results

### Engineering *E. coli dam *methyltransferase for expression in *C. elegans*

We first adapted the *E. coli dam *gene coding region for expression in *C. elegans*. We've previously found that introns incorporated into the coding regions can improve expression of transgenes in *C. elegans *[28]. The *dam *gene was cloned by PCR from *E. coli *strain OP50. Two introns were incorporated at blunt cutting restriction sites (Additional File 1, Figure S1). Two *myo-3 *promoter constructs driving *dam *were produced. In pPD177.01, DAM was designed to express as a fusion to GFP; while in pPD176.59, a nuclear localization signal (NLS) from SV40 was included. Each construct was then incorporated into transgenic strains using *pha-1*(+) as a selectable genetic marker in a *pha-1(e2123ts) *genetic background [29]. The two resulting transgenic lines were designated PD3994 (harboring pPD176.59) and PD5122 (harboring pPD177.01).

**Figure 1:**
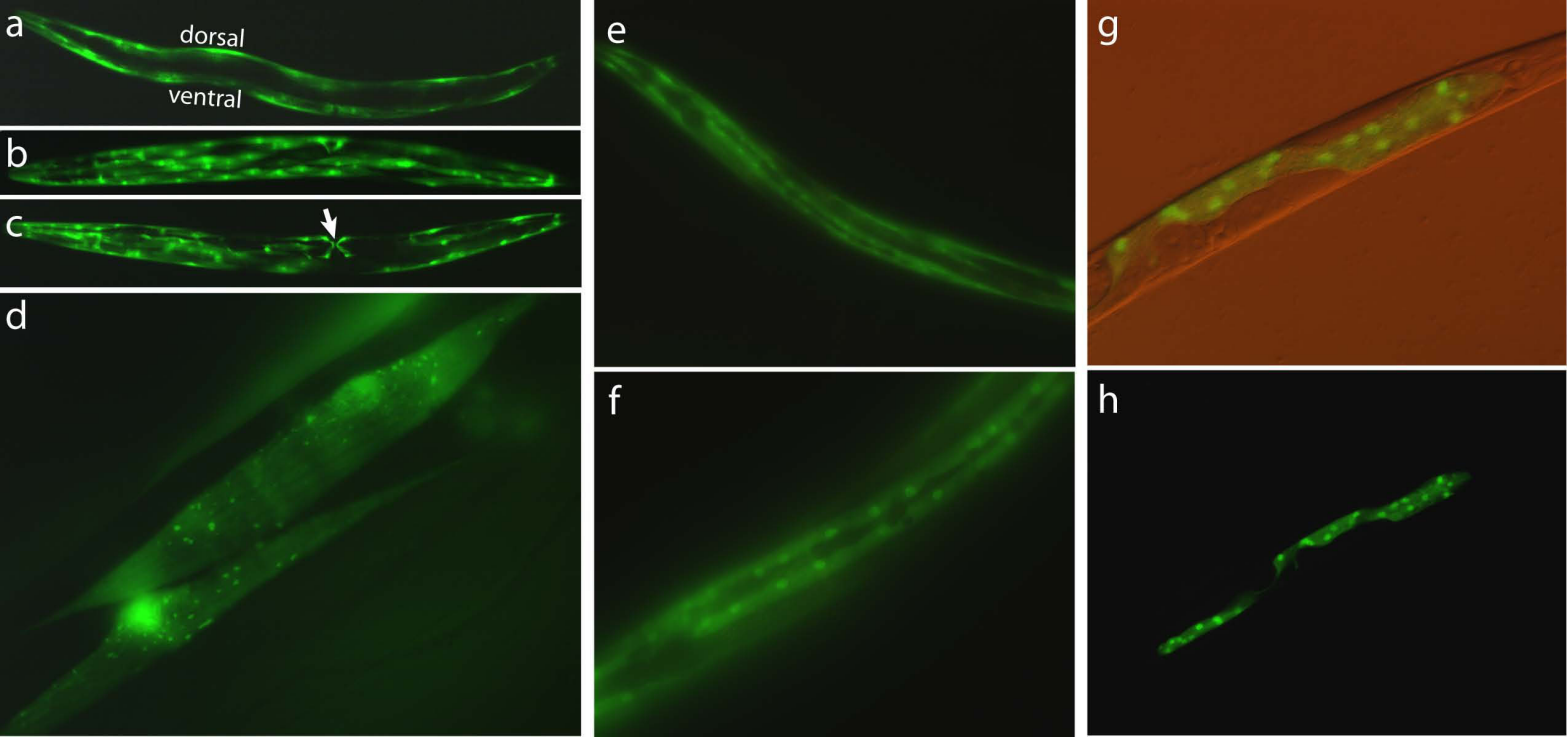
**Promoter specific expression of DAM methyltransferase in transgenic animals**. (a-d) PD5122 animals expressing DAM-GFP fusion driven by the *myo-3 *(body wall muscle) promoter. (a = L4,10X; b = adult,10X; c = adult,10X; d = adult,100X). (e-f) PD3995 animals expressing a DAM-GFP fusion construct driven by the *rol-6 *(hypodermal) promoter. (e = 200X; f = 400X). (g-h) PD3997 animals expressing a DAM-GFP construct driven by the *vit-2 *(gut) promoter. (g = 200X; h = 200X)

Both transgenic lines exhibited slightly slower movement and growth than wildtype animals; these effects were subtle, confounding any determination of whether this was due to the rescued *pha-1(e2123ts) *genetic background and/or expression of the methyltransferase. To assess tissue specificity of DAM expression, we examined PD5122 under fluorescence microscopy (Figure 1). When the animal is oriented laterally as in Figure 1a, the body wall muscles run along the animal's dorsal and ventral axes. A view from the animal's dorsal axis is shown in Figure 1b. In addition to body wall muscles, *myo-3 *is also expressed in the vulval muscles [28,30], easily identified as an "X" in the animal's ventral surface (Figure 1c, arrow). Within the body musculature, there is some degree of mosaicism in transgene expression in these animals. For example, notice in Figure 1c that certain body wall muscle cells (to the right and below the "X") lack GFP, possibly due to loss of expression or of the extra-chromosomal transgene array. We also note the presence of small GFP-positive granules in all these muscle cells (Figure 1d). We do not know the identity of these punctate structures, but we do not think they are mitochondria, as there are far more mitochondria that take up targeted GFP in *C. elegans *body wall muscle cells than what is seen in these pictures [31]. Since the DAM-GFP fusion used in PD5122 does not contain a characterized NLS, we were initially unsure whether the fusion protein would have access to the nucleus. As is evident in Figure 1d, not only did the methyltransferase have access to the nucleus, it appears to be predominantly found in the nucleus. Hypodermis-specific DAM-GFP expression from the *rol-6 *promoter (line PD3995) and gut-specific expression of DAM-GFP from the *vit-2 *promoter (line PD3997) are also evident (Figure 1 e,f and 1g,h). Together, these observations indicate that DAM can be engineered to express in a tissue-specific manner and that its localization to the nucleus can apparently occur without a NLS.

### DAM methyltransferase is active in *C. elegans*

**Figure 2:**
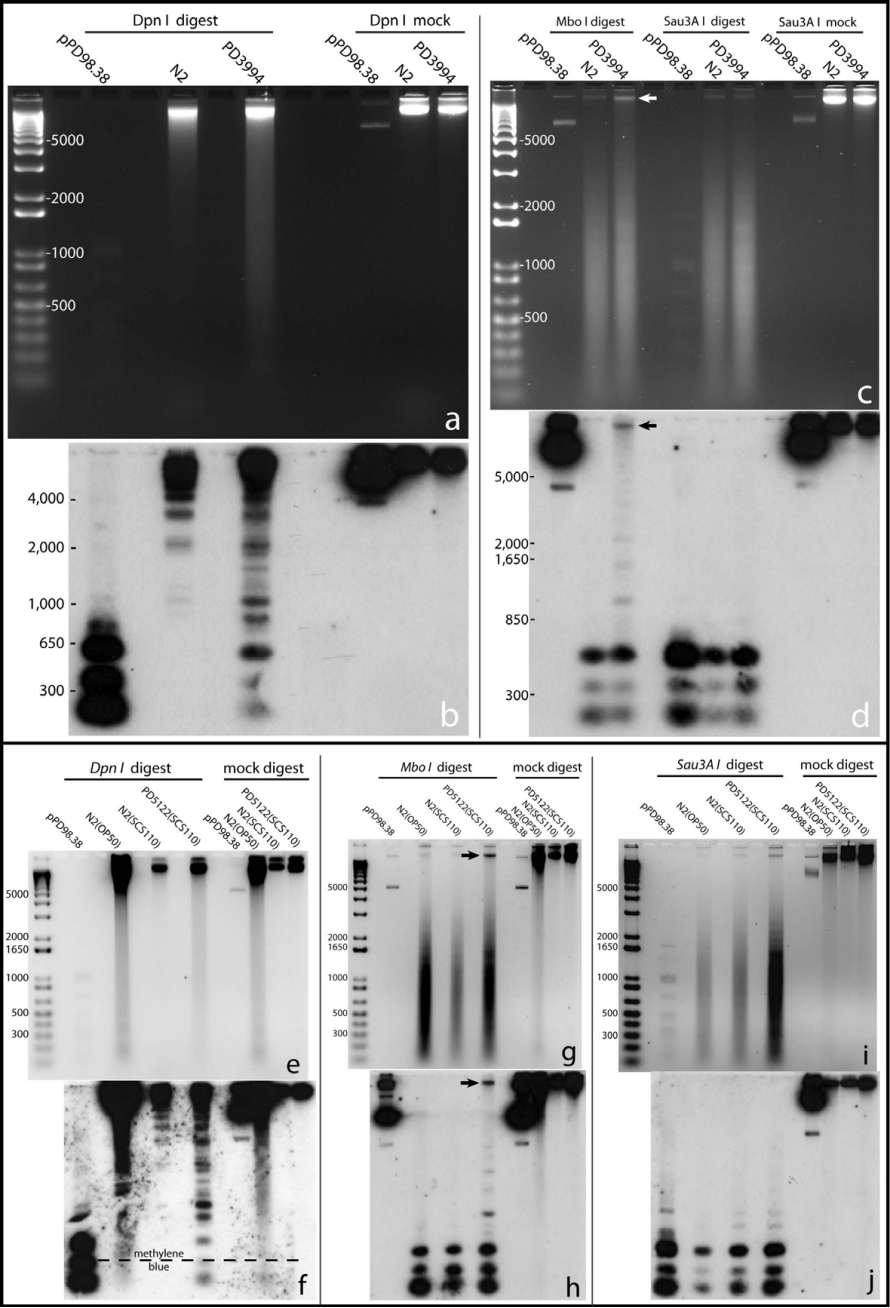
**Copy number determination for lines PD3994 and PD5122**. Panels a, c, e, g, and i are agarose gel images of *Dpn I*, *Mbo I*, and *Sau3A I *digested DNA from PD3994, PD5122, and N2 (control) animals. Below each agarose gel is the corresponding Southern blot (b, d, f, h, j). pPD98.38 is a plasmid from which the probe (808 bp of the *C. elegans *5S rDNA/SL1) was synthesized. The slight smearing seen in ethidium bromide stained gels for N2 (a and e) likely resulted from non specific activity of *Dpn I*. Compared to fully methylated GATC, *Dpn I *can cut non-methylated GATC 1,000 fold slower and hemimethylated GATC 60 fold slower (Derek Robinson, New England Biolabs, personal communication).

To assess the patterns of DAM methylation in transgenic animals, we used a combination of isoschizomer restriction enzymes that are differentially sensitive to GATC methylation. *Dpn I *cuts only GATC sites methylated on both strands; while *Mbo I *cuts only non-methylated GATC and *Sau3A I *cuts both methylated and non-methylated sites. Genomic DNA samples from wildtype (N2), PD3994, and PD5122 transgenic animals were digested with each restriction enzyme and probed with an ≈808 bp probe from the *C. elegans *5S/SL1 rDNA locus. The 5S/SL1 cluster consists of about 110 copies of a 1 kb repeat and generally has high transcriptional activity [32,33]. Southern blot analyses showed that the methylation status of the two DAM-expressing transgenic lines differed from that of the wildtype controls. The PD3994 and PD5122 lanes showed a strong smear that were nearly absent in N2, indicating that methylated DNA substrates were available for *Dpn I *restriction (Figure 2a,e). The smearing in the N2(OP50) lane in Figure 2e was likely due to *E. coli*(OP50) DNA from the animals' digestive tracts. Because the OP50 strain of *E. coli *carries the wildtype *dam *gene, its DNA is a substrate for *Dpn I *restriction.

The difference between DAM exposed and non-exposed DNA was clear in the corresponding Southern blots. Distinct *Dpn I *cleavage bands that were present in the PD3994 (Figure 2b) and PD5122 (Figure 2f) lanes were absent from the N2 lanes. An important feature of these gels is the appearance of bands indicative of incomplete digestion of individual fragments by *Dpn I*. Such bands were reproducibly observed in digestions of DNA from DAM-expressing lines (Figure 2b,f and data not shown), consistent with DAM modification of a subset (and not all) GATC sites in any given chromosomal molecule.

Restriction by *Mbo I *likewise revealed differences between DAM-exposed and wildtype DNAs. On bulk ethidium staining, genomic DNAs from PD3994 (Figure 2c, arrow) and PD5122 (Figure 2g, arrow) were left uncut by *Mbo I *compared to N2 DNA. The presence of considerable levels of digested DNA (smears in the agarose gels for PD3994 and PD5122) can be explained by the fact that the transgene arrays (*ccEx3994 *and *ccEx5122) *are driven by a muscle promoter and expressed only in muscle cells; non-muscle tissues (comprising about 90% of the body mass) would have been substrates for *Mbo I *restriction. In the Southern blots for *Mbo I *digests, (Figure 2d,h), wildtype DNA was essentially completely digested while DNA from transgenic animals were only partially digested. The unrestricted and partially restricted DNA (Figure 2d,h, arrows) would be expected to represent methylated DNA from muscle tissue. The ability of *Sau3A I *to cut GATC irrespective of DAM methylation provided a control for DNA quality and restrictability. As expected, identical *Sau3A I *patterns were observed in wildtype and DAM-expressing strains (compare *Sau3A I *lanes in Figs. 2c versus 2d, and 2i versus 2j). In combination, the Southern blot analysis demonstrates the ability of expressed DAM to modify the *C. elegans *genome in an extensive but limited manner.

We next carried out a pilot mapping of DAM methylation by conventional cloning and sequencing of *Dpn I *products. Each cloned fragment would have been expected to carry full methylation at both ends, with no methylation or hemi-methylation of intervening GATC sites which were not cleaved (Additional File 2, Figure S2). As a control, the cloning protocol was performed in parallel on wild type *C. elegans *DNA. Only small numbers of clones were recovered in this case, all derived from bacteria sequences or lacking the characteristic *Dpn I*-cleaved ends (data not shown). By contrast, large numbers of clones could be obtained from PD3994 and PD5122: 335 non-redundant *Dpn I *fragments were characterized from these (168 from PD3994 and 167 from PD5122), of which 314 had termini derived from *Dpn I *cleavage at methylated GATC sites (Additional File 2, Table S1). These sequences were distributed throughout the genome (Additional File 2, Figure S3) and spanned exons, introns, exon-intron junctions, and non-annotated (intergenic) regions (Additional File 2, Tables S2 and S3). In addition to the genomic distribution, it was of interest to observe fragments from muscle-expressed and non-muscle expressed genes (Additional File 2, Tables S4 and S5).

### Profiling genome-wide chromatin accessibility using Direct Asymmetric Ligation End Capture (DALEC)

**Figure 3:**
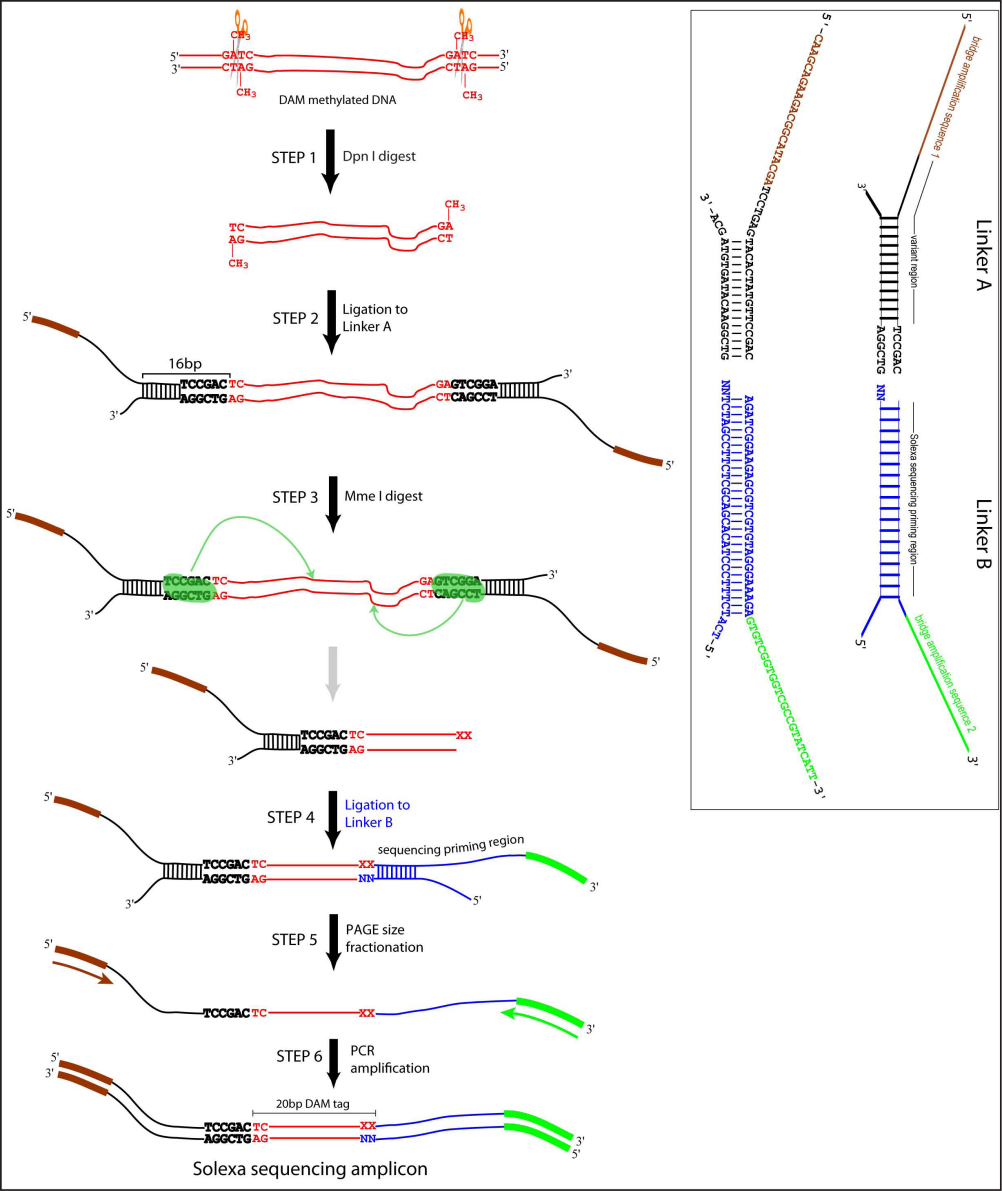
**Schematic of DALEC**. Brown and green represent priming regions for Solexa bridge amplification primers. Blue indicates the primer binding region for the Solexa sequencing reaction. TCCGAC is the *Mme I *recognition sequence and the sequence immediately upstream of it (black) is the variable region. The inset gives detailed information for linkers A and B.

To obtain a genome-level view of chromatin susceptibility to DAM methylation, we developed a method that captures sequences flanking methylated GATC sites for high throughput sequencing (Figure 3). Genomic DNA from transgenic animals expressing tissue-specific DAM is digested with *Dpn I*. The resulting blunt-ended *Dpn I *product is captured by ligation to a branched linker ("A", inset) containing an asymmetric restriction site (*Mme I*) adjacent to the site of ligation. *Mme I *cleavage of these ligation products liberates a population of fragments containing ≈20 bp of *C. elegans *DNA with a two nucleotide 3" overhang (Step 3 product). The *Mme I *product is ligated to a second branched linker ("B", inset), consisting of a pool of 16 different species of molecules that differ only at the 3" two nucleotide overhang. The top strand (116 nucleotides long) of the resulting doubly-linkered molecule is separated from the bottom strand (71 nucleotides long) using denaturing polyacrylamide gel electrophoresis and amplified by PCR using Solexa bridge amplification primers [5'-AATGATACGGCGACCACCGA-3', 5'-CAAGCAGAAGACGGCATACGA-3']. To achieve quantitative results, we titrated the PCR reactions to ensure amplification in the linear range. In our experience, typically 11-15 cycles are sufficient. The appearance of a band migrating at or near 125 bp (in addition to the 116 bp band) is diagnostic of over-amplification. The PCR product is electrophoresced in a 3% low melting point agarose gel (i.e. NuSieve™) and the 116 bp band excised and purified. The 116 bp amplicon is directly sequenceable on the Illumina platform. (See Additional File 2 Methods section for additional notes on DALEC assay).

We constructed and sequenced libraries from animals expressing *myo-3*::*dam *(PD3994, muscle), *rol-6*::*dam*-GFP (PD3995, hypodermis) and *vit-2*::*dam*-GFP (PD3997, gut), with a control library from N2 DNA treated *in vitro *with DAM methyltransferase. Animals were staged to maximize the mass of the tissue expressing the DAM protein (L1 for muscle; L4 and young adults for hypodermis; adults for gut). A combined total of 28.1 million raw reads were obtained from two separate sequencing runs. Linker sequences could be successfully parsed out from 25,319,003 reads. We considered a parsing event successful if the resulting product had the structure 5'-N_16-19_GA-3'. Parsed readouts were in turn converted to a 16 nucleotide format containing only the 16 nucleotides immediately upstream of the 3' GA. Parsed tags were then mapped to a database containing all filtered DAM tags in the *C. elegans *WS170 genome. A "hit" was considered only if there was a 16 nucleotide perfect match between the Solexa readout and the *in silico*-generated tag. Since each 16 nt tag in the database actually represented a molecule of the structure 5-(N)_16_GATC-3', our criterion in actuality required a 20 nt perfect match. In total, 11,229,470 reads could be aligned to the genome. After exclusion of repetitive and "proximal" tags (see Additional File 2, Figure S4 for definition and filtering of "proximal" tags), we obtained 9,651,128 tags that aligned uniquely to the genome, representing an average of 9-fold coverage (per GATC site in the diploid genome). These were used for all analyses described below.

### Relationship between expression and DAM accessibility

**Figure 4:**
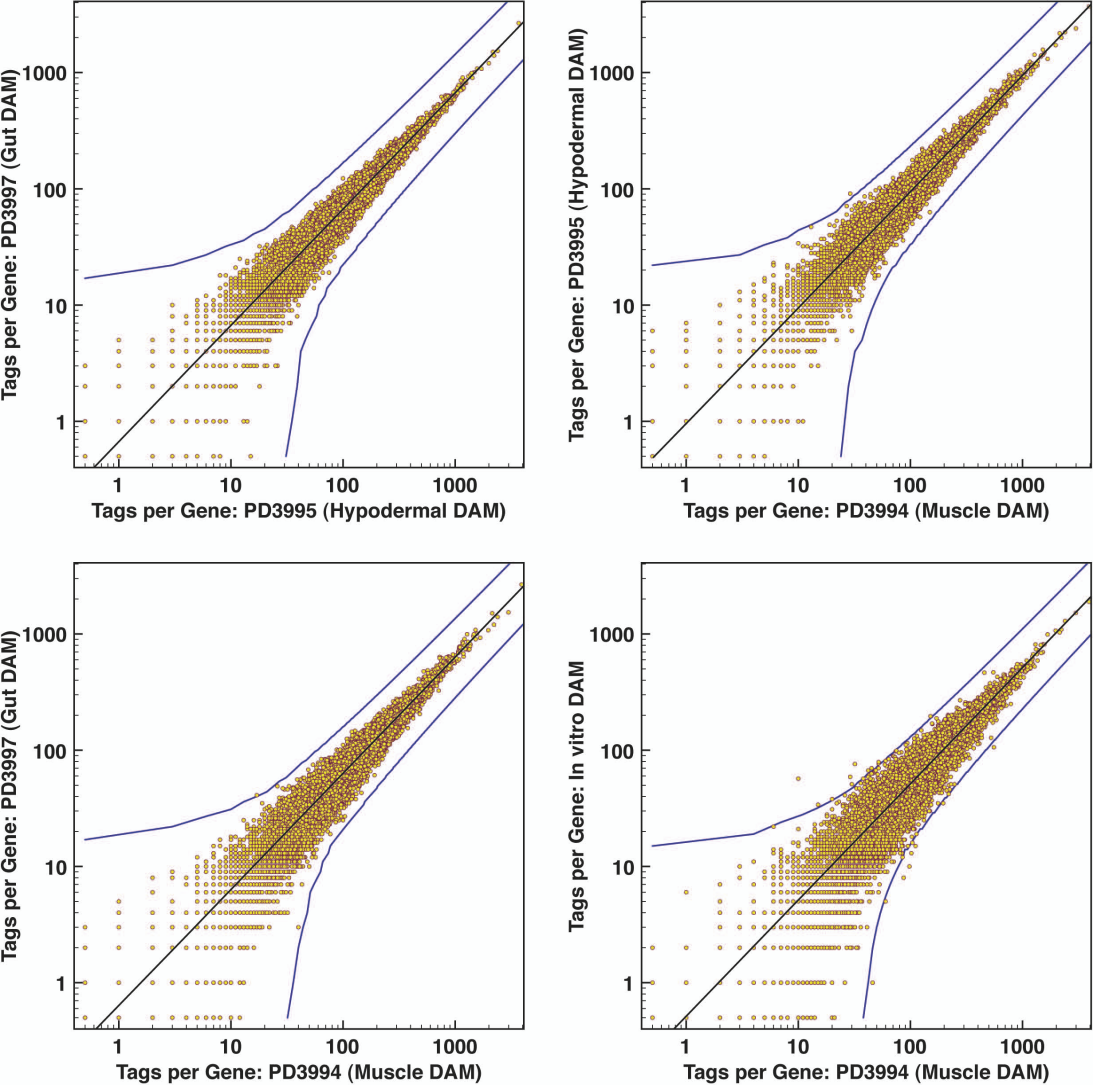
**Minimal global inaccessibility of inactive genes in differentiated tissues**. Each point represents a gene. The X and Y axes indicate promoter-specific DAM accessibility across the two tissues being compared. The bottom right panel is the comparison between *in vitro *methylated N2 DNA versus DAM-exposed DNA from muscle tissue. The black line is normalized equivalence; while the blue curves demarcate the level of divergence that is (1) at least two-fold, and (2) statistically significant to P < 0.05

The analysis of DAM-DALEC data for different tissues provides an opportunity to characterize the relationship between gene activity and DAM accessibility. We first compared total numbers of DAM "hits" between individual genes in pairs of samples from the analysis. Raw tag numbers for each gene in any two data sets were used as X and Y values to place a point on a graph, so that a neutral situation in which tags were equivalently distributed between samples would produce points along a straight line. A striking feature of this analysis was the remarkable concordance between samples in representation of tags for individual genes (Figure 4). Despite the tissue-restricted expression of many genes in these individual tissues [34], a significant deviation in overall DAM accessibility was essentially limited to a two-fold range. As a control, we examined DNA segments present in the transgene constructs (and thus at higher copy numbers) in the individual lines; this analysis showed the expected substantial differences in tag representation between samples (data not shown). A similar comparison for functional partitions of the genome (introns, exons, and sequences flanking the annotated gene boundary) is presented in Additional File 2, Figure S5. As with the gene-by-gene comparisons, we did not observe regions with a greater than two-fold deviation from the mean for the genome.

**Figure 5:**
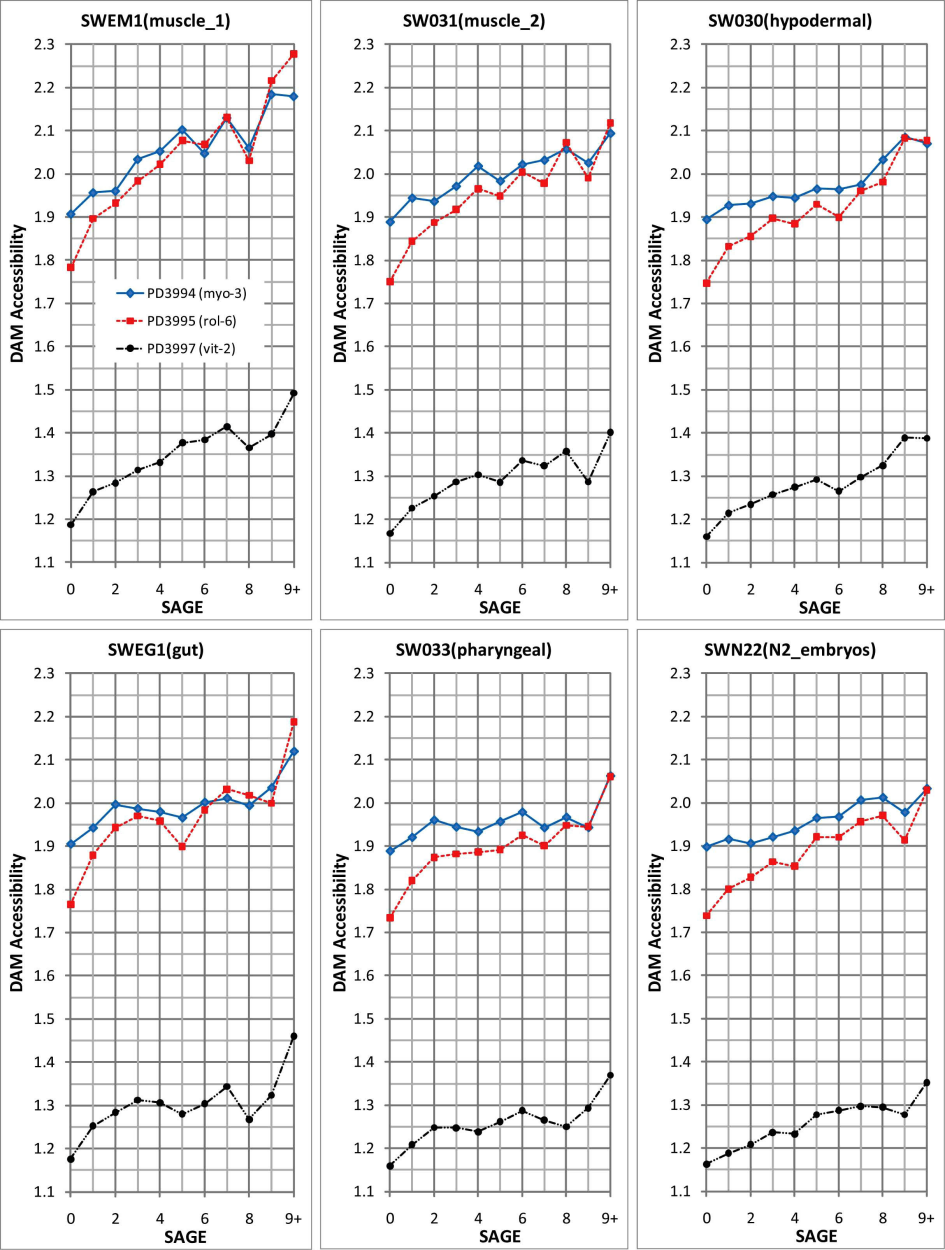
**DAM accessibility correlates with SAGE expression levels**. X-axis indicates SAGE scores (i.e. SAGE hit counts). Y-axis indicates DAM accessibility index (ratio of tissue-specific DAM hits normalized over control genomic DNA DAM hits). Only tags that had at least four hit half sites (where each half site must have been hit at least once for any one of the tissue samples *myo-3*, *rol-6*, *vit-2*, and genomic DNA control) were used in the analysis. Following a calculation for each sample of the average number of hits per GATC site (using the tags' "txStart" and "txEnd" from the UCSC genome browser version WS170), the final DAM accessibility index for a particular tissue was determined by normalizing average hits per GATC to that observed for the *in vitro *DAM treated *C. elegans *genomic DNA control. Each point represents the average of the accessibility indices of all genes that have the same SAGE score. The "9+" category represents the aggregate average of all DAM accessibility indices with SAGE scores greater than 9.

Despite the lack of large deviations from a mean accessibility, we do observe genes and genomic segments with some significant deviation from the mean in their tag representation in individual samples. To determine potential relationships between variations in accessibility and expression, we compared accessibility indices as a function of gene representation in a filtered SAGE dataset (Figure 5; see Materials and Methods section for SAGE filtering parameters and analysis methodology). Notably, the magnitudes of the differences are rather modest (less than 1.25-fold). Nonetheless, this analysis shows a reproducible positive correlation between average DAM accessibility and gene expression for all three transgenic lines.

### A periodic DAM accessibility profile that correlates with nucleosome positioning at promoters

To characterize chromatin structure and accessibility on a subgenic level, we compared DAM methylation profile with previously published nucleosome position datasets for *C. elegans*. Of particular interest are datasets of total nucleosome positioning [35] and those in which a nucleosomal population was enriched for active chromatin by immunoprecipitation using antibodies to modified histones, in particular methylation on lysine 4 of histone H3 [36]. The H3K4me2/3 nucleosome occurs at the 5' end of actively expressed genes and displays a high degree of constraint and phasing characteristics of its positioning, with bulk nucleosomes showing a much lower degree of reproducibility in positioning [35]. A prominent "peak" H3K4me2/3 nucleosome can be readily detected at the 5' end of 3,904 genes, the majority of which are house-keeping genes (e.g. ribosomal proteins). To represent the relationship between nucleosome position and DAM accessibility, we calculated *n *numbers of DAM tags each position (relative to the peak dyad, normalizing to the number of DAM sites at the same position). A signal in this analysis depends on significant local positioning of nucleosomes, and not surprisingly we obtained little signal with the less-positioned bulk nucleosomes (data not shown).

**Figure 6:**
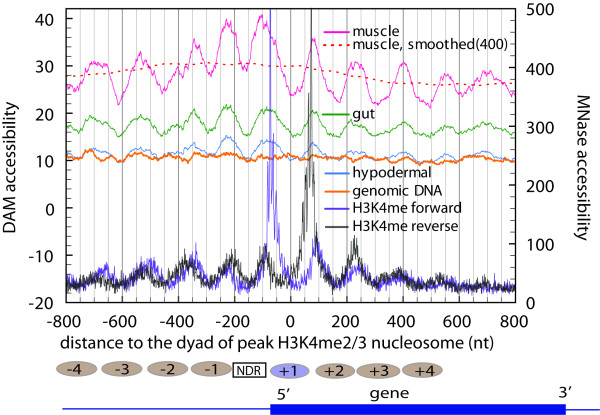
**DAM accessibility correlates with nucleosome positions**. This figure shows the superimposition of average DAM accessibility versus average MNase accessibility around the transcription start site (TSS) of 3,904 strongly expressed *C. elegans *genes. The dotted red curve represents a moving average of the muscle profile with a sliding window of 400 nucleotides. Positioned H3K4me2/3 nucleosomes are represented by ovals above the picture of the generic gene. Numbers on the nucleosomes indicate their positions relative to the TSS. [NDR = nucleosome depleted region]

For the H3K4 methylated nucleosomes, we saw a distinctive pattern (Figure 6), with the overall DAM methylation levels oscillating between a valley at a nucleosome dyad position and a peak at linker region. The concordance is especially strong for muscle (pink) and gut (green). The somewhat low concordance for hypodermal (blue) is most likely the result of low tag representation (due to less input DNA for library preparation). The same analysis on the control *in vitro *modified genomic DNA displayed a profile that was essentially flat. This result is consistent with the preference of linker DNA over nucleosome core DNA for DAM methylation. As an independent probe, DAM methylation confirms the nucleosome positioning near 5' end of genes that have been characterized by using micrococcal nuclease digestion.

Regions that are upstream to the H3K4me2/3 peak nucleosomes have been found to display a low level of observed nucleosome coverage. Correspondingly, the overall DAM methylation level is higher in regions upstream than in regions downstream to the H3K4me2/3 peak nucleosome.

Nucleosome coverage at ≈150bp upstream ("NDR" in Figure 6) to the dyad of the H3K4me2/3 peak nucleosome has a lowest level. If DNA in this region tends to be in an unprotected state, one would expect to observe a high level of DAM accessibility. Instead, there is a valley at this position for DAM methylation profiles from all three tissues. This result suggests that DNA in this region is protected by additional factors.

## Discussion

In this article, we presented our study of chromatin structure in differentiated *C. elegans *tissues by measuring DNA accessibility in living animals. To examine a series of *in vivo *accessibility profiles, we expressed *dam *driven by three tissue-specific promoters (*myo-3*, *rol-6*, *vit-2*) and analyzed the methylation profile of synchronized animal populations. Such an analysis would be expected to identify both dramatic and subtle differences in *in vivo *accessibility. Somewhat surprisingly given a number of models for developmental regulation that involve higher order inaccessibility of large regions of chromatin [37], we observed no genes or regions which were completely inaccessible to DAM modification *in vivo*. In particular, no region or gene showed a deviation in accessibility that was greater than from the genome-wide average. Analysis of quantitative differences in DAM accessibility does reveal correlations, particularly showing a relationship between accessibility and expression levels: with increasing SAGE representation numbers, we observe a corresponding increase in accessibility over many genes and in all samples and tissues analyzed. Because SAGE measures expression by capturing the pool of mature mRNAs, our results suggest that DAM accessibility at least partially reflects average transcriptional activity.

Although the data argue against "open/shut" accessibility versus inaccessibility of non-expressed chromosomal domains during development, there is certainly evidence for stable structures that protect specific sequences for extended time periods. These are evidenced by the capture of *Dpn I *fragments with internal (non-methylated) GATC sites (Additional File 2, Figure S2 and Table S1). These internal sites would have been protected from DAM methylation for an extended period while DAM was expressed in the relevant cells.

We observed very strong correlation between DAM accessibility and nucleosome positioning, in agreement with previous work [4]. As shown in Figure 6, DAM accessibility peaks at inter-nucleosomal regions, indicative of the accessibility of linker DNA. As the nucleosomes become less uniform in position (beyond 500 bp upstream and downstream of the TSS), the periodicity in DAM methylation profile is decreased.

Our results provide support for DAM-DALEC and nucleosome ChIP-Seq [36,38] as complementary technologies in establishing and validating detailed chromatin maps. Certainly ChIP-Seq provides the highest resolution maps for chromatin; at the same time this technique is limited by the need for extensive processing of samples after chromatin extraction. DAM-DALEC provides an *in vivo *picture of chromatin structure that is unaffected by concerns of specificity and rearrangement on extraction but is lower resolution in terms of the numbers of sites analyzed. Certainly the combination of the two methods will be of value in defining both static architecture and developmental shifts.

## Conclusions

We have developed an assay with the capacity to infer chromatin structure on a genome level in living organisms. We have shown its concordance to gene expression and positioned nucleosome data obtained from independent sources. Thus, DAM-DALEC can provide independent high-confidence *in vivo *data, which even for a fraction of the genome can be used to refine, validate, or evaluate less sparse but potentially more 'invasive' nuclease-based assays. DALEC could readily be adapted to any context in which expression of foreign coding regions (*dam *methyltransferase) can be engineered. Because eukaryotes do not possess an adenine methylation system, the enzyme would have a possibility of being "neutral" to the host cell and not subject to regulation. Thus, DAM-DALEC could offer an advantage of capturing snapshots of chromatin structure in living animals at defined developmental stages and can be a powerful tool that complements existing genomics methods for investigating chromatin structure and dynamics on a whole genome level.

## Methods

### *C. elegans *strains and growth conditions

Animals were reared on *E. coli *grown on NGM (nematode growth medium) nutrient plates [39]. Bacterial strains used in this work as food for *C. elegans *are noted for each experiment.

**OP50**[39]: A uracil-auxotrophic *E. coli *strain with wild-type *dam *and *dcm *methylation systems. This strain has been a standard laboratory food source for *C. elegans*.

**SCS110:** An *E. coli *strain defective in both *dcm *and *dam *methylation: *rpsL *(Str^r^) *thr leu endA thi-1 lacY galK galT ara tonA tsx dam dcm supE44D *(*lac-proAB*) [F' *traD36 proAB lacI*^*q*^*ZDM15*]. This strain provides a suitable food source for *C. elegans *while avoiding the presence of bacterial sequences with adenine methylation in eventual sequencing libraries.

**SCS110(Amp^R^):** SCS110 made ampicillin resistant by transformation with pUC18. This strain adds the ability to "switch" bacterial food sources in a culture by cultivation of previously OP50-fed populations with SCS110(AmpR)^+ ^and ampicillin.

All worm strains were reared at 23°C unless otherwise stated. *C. elegans *strains used in the experiments were as follows:

**N2:** wildtype strain of *C. elegans *(Bristol isolate)[39]

**PD5122 [*pha-1*(*e2123ts*) III; *ccEx5122*]:** transgenic line expressing *E. coli dam*-GFP translational fusion and genomic *C. elegans pha-1*(+) gene from the extra-chromosomal array *ccEX5122*. Line PD5122 was established by microinjection of a mixture of plasmids pPD177.01 (Lig6682) and pC1 into *pha-1*(*e2123ts*) animals. pPD177.01 contains the *myo-3 *(body wall muscle) promoter driving *E. coli dam *fused to GFP. Two introns with *C. elegans *consensus sequences have been inserted into the *dam *gene to optimize expression in nematodes [28,40]. A detailed description of pPD177.01 structure is shown in Additional File 1, Figure S1. pC1 carries the wildtype *pha-1 *coding region; non-transformed *pha-1*(*e2123ts*) animals are inviable at 23°C, while transformed animals carrying pC1 are viable, providing a strong selection [29].

**PD3994 [*pha-1*(*e2123ts*) III; *ccEx3994*]:** transgenic line expressing *E. coli dam *and genomic *C. elegans pha-1*(+) gene from the extra-chromosomal array *ccEx3994*. Line PD3994 was established by microinjection of plasmids pPD176.59 (Lig6649) and pC1(FD142), which contains the *C. elegans *genomic *pha-1 *gene [29] and is a selection marker for *ccEx3994*. pPD176.59 contains the *E. coli dam *gene driven by the *myo-3 *promoter and a single SV40 nuclear localization signal.

**PD3995 [*pha-1*(*e2123ts*) III; *ccEx3995*]:** transgenic line expressing *E. coli dam *and genomic *C. elegans pha-1*(+) gene from the extra-chromosomal array *ccEx3995*. Line PD3995 was generated by microinjection of a mixture of plasmids L7710, pRF4 (carrying the *C. elegans rol-6*[*su1006*] [41]), and pC1 into *pha-1*(*e2123ts*) animals. Plasmid L7710 contains the *rol-6 *promoter [42] driving the expression of a GFP-DAM translational fusion (*rol-6*::*gfp-dam*-*unc-54 *3' UTR). Attached to the 3' end of L7710 is the *unc-54 *3' UTR [28].

**PD3997[*pha-1*(*e2123ts*) III; *ccEx3997*]:** transgenic line expressing *E. coli dam *and genomic *C. elegans pha-1*(+) gene from the extra-chromosomal array *ccEx3997*. Line PD3997 was established by microinjection of a mixture of plasmids consisting of pC1, pRF4, and L7715 (*vit-2*::*gfp-dam-unc-54 *3' UTR).

### Southern hybridization

Southern hybridizations were performed according to standard protocols. Briefly, RNase A-treated genomic DNA was subjected to one hour restriction digest by *Dpn I*, *Mbo I*, or *Sau3A I*, followed by phenol:chloroform extraction and ethanol precipitation. Restricted fragments were resolved on 1.4% agarose gels followed by transfer to Hybond-N+ membranes (Amersham Biosciences, Cat #RPN303B as recommended for capillary blotting under alkali conditions). An 808 bp radiolabeled probe containing the *C. elegans *5S rDNA/SL1 (Spliced Leader sequence 1) was synthesized from a *Bam H1*fragment of plasmid pPD98.38 using the RadPrime DNA Labeling System (Invitrogen, Cat #18428-11) with labeled α-^32^P dATP (MP Biomedicals, Cat #33002HD.5). Pre-hybridization and hybridization were in roller bottles using phosphate-SDS buffer (0.5 M phosphate buffer pH7.2, 1 mM EDTA pH8.0, 7% (w/v) SDS, 1% (w/v) BSA).

### DNA extraction from synchronized animal populations

To generate synchronized worm populations, embryos were collected by treating gravid animals with a solution containing 1 M NaOH in 10% bleach for approximately 5-7 minutes or until adult cuticles were completely disintegrated [39]. Eggs were washed several times with M9 medium (22 mM KH_2_PO_4_, 42 mM Na_2_HPO_4_, 86 mM NaCl, 1 mM MgSO_4_) and distributed onto NGM plates containing a thin layer of SCS110 *E. coli *seeded the previous night. Synchronized populations were collected at the stage where the desired tissue mass would be greatest per animal (L1/L2 larvae for the *myo-3 *promoter in line PD3994; L4 larvae for the *rol-6 *promoter in line PD3995; and young/gravid adults for the *vit-2 *promoter in line PD3997). At no time were synchronized animals starved.

Synchronized animals were washed off NGM plates with chilled M9 medium, layered on a 5% sucrose solution, and pelleted by centrifugation at low speed. Pelleted animals were washed several times with chilled M9 and frozen as ~50 μL pellets at -80°C. It is important to note that throughout the harvesting procedure, animals were alive up to the time before freezing.

Genomic DNA was extracted using the following procedure. To each thawed ≈50 μl pellet was added 450 μl worm lysis buffer (0.1 M Tris pH 8.5, 0.1 M NaCl, 50 mM EDTA, 1% SDS), 1 μl 10 mg/ml glycogen, and 20 μL 20 mg/ml proteinase K in TE pH 7.4. The mixture was incubated at 62°C for 45 minutes, with intermittent vortexing. The mixture was extracted with 500 μl phenol, followed by 500 μl phenol:chloroform (1:1), and 500 μl chloroform. DNA was precipitated with 20 μl saturated ammonium acetate and 1 ml 100% ethanol, washed once with 500 μl ethanol, and resuspended in 50 μl TE pH 7.4. Each 50 μl sample was treated with 1 μl 10 mg/ml RNase A for one hour at 37°C. The reaction was terminated with 1× STOP buffer (1 M NH_4_Ac, 10 mM EDTA, 0.2% SDS) followed by phenol:chloroform/chloroform extraction and 100% ethanol precipitation. The final product was resuspended in 40-50 μl TE and used for DALEC library preparation.

### *in vitro *methylation of N2 genomic DNA

N2 genomic DNA was methylated using the following 200 μl reaction mixture: 30.0 μl (≈20-30 μg) N2 genomic DNA, 0.5 μl 32 mM S-adenosyl methionine, 1.0 μl *E. coli *DAM (8 U/μl, NEB M0222S), 20.0 μl 10× DAM buffer, 148.5 μl dH_2_O. Following one hour incubation at 37°C, the reaction was terminated with 1× STOP buffer. To the terminated reaction mixture was added 1 μl 10 mg/ml glycogen followed by 500 μl phenol:chloroform extraction, 500 μl chloroform extraction, 100% ethanol precipitation, 0.5 ml 100% ethanol wash.

### *Dpn I *digestion

*Dpn I *digestion was carried out in a 200 μl volume consisting of the following mix: 30 μl (≈20-30 μg) genomic DNA, 20 μl 10× buffer (NEB4), 10 μl *Dpn I *(20 U/μl, NEB #R0176), and 140 μl dH_2_O. The reaction mix was incubated for 1.5 hours at 37°C and terminated with 350 μl 1× STOP buffer. 1.0 μl 10 mg/ml glycogen was added to the mix followed by 500 μl phenol:chloroform extraction, 500 μl chloroform extraction, precipitation with 100% ethanol, wash with 0.5 ml 100% ethanol, and resuspension in 10 μl TE. (NOTE: Unless otherwise indicated, all enzymatic reactions described below used the same termination, extraction, and precipitation steps).

### Ligation to Linker A

Linker A was purchased as two separate oligonucleotides (5'OH-CAAGCAGAAGACGGCATACGATCCTGAGTACACTATGTTCCGAC-OH3', 5'P-GTCGGAACATAGTGTAGCA-OH3') and hybridized by boiling in a flask of water for five minutes and allowing the water to cool to room temperature. Ligation to Linker A was carried out in a 50 μl reaction using the following mix: 10 μl *Dpn I *product, 1.5 μl of 0.05 mM Linker A, 11.0 μl dH_2_O, 25.0 μl 2× Quick Ligase Buffer, 2.5 μl Quick Ligase (NEB #M2200). The reaction was incubated for five minutes at room temperature followed by termination, extraction, precipitation, and resuspension of ligated products in 10 μl TE.

To increase the number of ligated molecules, we added a second ligation step using the following mix: 10 μl Quick Ligase product, 7 μl dH_2_O, 2 μl 10× ligase buffer, 1 μl T4 DNA ligase (2,000 U/μl; NEB #M0202). The reaction was allowed to proceed for 30 minutes at room temperature followed by termination, extraction, precipitation, and resuspension in 20 μl TE.

### *Mme I *digestion

Linker A-ligated molecules were subjected to *Mme I *digestion using the following 200 μl reaction mix: 20 μl Linker A-ligated product, 20.0 μl 10× NEB 4, 0.3 μl 32 mM S-adenosyl methionine, 2.0 μl *Mme I *(2 U/μl; NEB R0637), 157.7 μl dH_2_O. The reaction was allowed to proceed for 1 hour at 37°C followed by termination, extraction, precipitation, and resuspension in 10 μl TE.

### Ligation to Linker B

Linker B was purchased as two separate oligonucleotides (5'P-AGATCGGAAGAGCGTCGTGTAGGGAAAGAGTGTCGGTGGTCGCCGTATCATT-OH3', 5'OH-TCATCTTTCCCTACACGACGCTCTTCCGATCTNN-OH3') and hybridized using the same procedure as described for Linker A. *Mme I *products were ligated to Linker B using the following 50 μl reaction mix: 10.0 μl *Mme I *product, 1.0 μl of 0.05 mM Linker B, 5.0 μl 10× ligase buffer, 3.0 μl T4 DNA ligase (2,000 U/μl; NEB #M0202), 31.0 μl dH_2_O. Ligations were performed overnight using a PCR machine. Reactions were initiated at 8°C and stepped up to 16°C, with each degree increase in temperature held for two hours. Ligated products were extracted, precipitated, and resuspended in 10 μl TE.

### Size selection

Linker A and Linker B ligated molecules were size fractionated on a 6% polyacrylamide:formamide denaturing gel (15% v/v 19:1 acrylamide:bis (40%), 1× TBE, 25% (v/v) 100% formamide, 42% w/v urea). Electrophoresis was performed in 0.5× TBE at 700 V for approximately 2.5-3 hours. The 116 nt single-stranded DNA product was cut out from the denaturing gel, using single-stranded oligonucleotides of sizes 95, 105, 114, 116, and 125 as size guides. Products were passively eluted from excised bands overnight in 0.3 M NaCl at 4°C, precipitated in 100% ethanol, and resuspended in 20 μl TE.

### PCR amplification

PCR reactions were performed in a 50 μl reaction consisting of the following mix: 5 μl PAGE-purified template, 1.0 μl each of Solexa bridge amplification primers (1 μg/μl), 5 μl dNTP mix (2 mM each), 5 μl 10× NEB ThermoPol PCR buffer, 1 μl Taq polymerase (5 U/μl; NEB M0267), 32 μl dH_2_O. Reaction cycles were titrated to determine the linear range, typically 11-17 cycles of 45 s at 94°C, 30 s at 55°C, and 30 s at 72°C, with an initial denaturation step of 60 s at 94°C and a final extension step of 60 s at 72°C. PCR products were separated on 3% low melting point agarose gel (NuSieve Cat #50084). For a gel of approximately 10 inches long, electrophoresis at 103 V for approximately six hours gave superior resolution. The desired 116 bp dsDNA product was excised and recovered from the gel using the following steps. To each cut band was added 400 μl 1× STOP buffer, 1 μl glycogen and incubated in a 68°C water bath until the agarose was completely melted. To each tube of melted agarose was added 350 μl of 68°C phenol, quickly vortexed, spun 5-7 minutes, and the aqueous phase extracted (typically, a second 1-2 minute spin was required to completely remove residual agarose). Following extraction with 250 μl 1:1 phenol:chloroform and 250 μl chloroform, DNA was precipitated in 1 ml 100% ethanol, washed with 0.5 ml 100% ethanol, and resuspended in 15 μl TE for each 5 μl PCR template used.

### Sequencing

Sanger sequencing was performed by Elim Biopharmaceuticals Inc. (Hayward, CA). High throughput sequencing of captured DAM tags was performed on the Solexa Genome Analyzer I.

### *in silico *identification of DAM tags

We generated a database of all potential DAM tags with the structure 5'-(N)_16_GATC-3' from *C. elegans *genome version WS170. There are 269,049 DAM (GATC) sites per haploid genome in *C. elegans*. Because DALEC captures two tags (in principle) per GATC site, there are a total of 538,098 potential tags (or half sites) per haploid genome. To reduce computation time during alignment of Solexa reads to the genome, each tag was represented by a 16 nucleotide sequence that did not include the 3' GATC.

We excluded DAM tags that occurred more than once in the genome or that mapped to vector or ribosomal sequences. We also excluded tags belonging to two adjacent GATC sites that lie within 20 bp from each other. Under situations where two fully methylated adjacent GATC sites mapped within 20 bp of each other, one site will always be captured at the expense of the other, resulting in undercount of DAM accessibility at such regions. When the distances are slightly above 20 bp, it is conceivable that there may be inherent bias in *Mme I *sequence preference that leads to the preferential capture of one site over the other, again resulting in undercount. To avoid both situations from skewing our analysis, we excluded such "proximal tags" using the criteria described in Additional File 2, Figure S4). After filtering out proximal, repetitive, and vector/ribosome-derived sequences, we were left with 370,152 *in silico *tags (per haploid genome) that we could use to align Solexa reads to the genome.

### SAGE analysis

SAGE data were obtained from the Genome BC *C. elegans *Gene Expression Consortium at http://elegans.bcgsc.bc.ca/[43]. We downloaded the March 2006 *C. elegans *SAGE database using the following relatively standard parameters: Quality filter: 0.99, Hide ambiguous tags: ON, Tag mapping resources: CODING, show only mapped tags: ON, Tags/page: 10, Lowest count cutoff: 1, Hide antisense tags: ON, Remove duplicate ditags: ON, Highest count cutoff: NONE, Sort order: DOWN, Resolve lowest match: ON. We used only long SAGE tags (17 nucleotides long) in our analysis. To determine a total SAGE score for each gene, we collapsed redundant annotations for each gene to a single copy and summed the SAGE score for each annotation. After our filtering criteria, we were left with 13,916 unique genes in our SAGE data set.

All data sets, including raw and aligned Solexa reads, SAGE data sets, *in silico *generated Dam tags, and gene sets used in our analyses have been deposited into GEO with accession number GSE23042.

## Abbreviations

DALEC: Direct Asymmetric Ligation End Capture; DAM: DNA Adenine Methyltransferase; GFP: Green Fluorescent Protein; H3K4me2/3: di- or tri-methylation of lysine 4 on histone H3; MNase: micrococcal nuclease; NDR: Nucleosome Depleted Region; NEB: New England Biolabs; SAGE: Serial Analysis of Gene Expression

## Competing interests Statement

The authors declare that they have no competing interests.

## Authors' contributions

KS, LCP, and AF designed the research project. KS, SGG, and AF designed and implemented analytical tools. KS, LCP, AG, JF, DB, and CK designed and carried out experiments. KS, SGG, and AF wrote the manuscript. All authors have read and approved the final manuscript.

## Supplementary Material

Additional file 1 **Sequence of plasmid pPD177.01 in Microsoft Word format**. Click here for file

Additional file 2 **Supplemental materials, including notes on methods and supplemental figures and tables in Microsoft Word format**. Click here for file
