# Supplementary material for: Distributed probing of chromatin structure in vivo reveals pervasive chromatin accessibility for expressed and non-expressed genes during tissue differentiation in C. elegans
Source: BMC Genomics. 2010 Aug 6;11:465. doi: 10.1186/1471-2164-11-465 (PMC3091661; doi:10.1186/1471-2164-11-465)
Supplement: Additional file 1 — Sequence of plasmid pPD177.01 in Microsoft Word format. [file 1471-2164-11-465-S1.PDF]

HindIII *myo-3* **5' Flank and Trans Splice Acceptor**
aagcttggggctgcaggtcggctataataagttcttgaataaaataatttt
cccgacaaaacatgagtatttctttcgaaaataaaagtgcaggctaatta
gagattattctgtaattaactgcataatttgtcacgtgccatagttttac
attccactacgtcatagttcttaaaatactaatctcctgaaaatagaagt
aggtgagaaagtttaattatcagttctaaaatgacaattgatctttgga
atatgttctgaaactaccgatcattgaacagatgctatttgaatgatata
gaattgtatatttgcaatttctgaaacgcgttcttaaaggcacacagatt
aattcaaaagggtctggccgcaaaaaggtttatggtggccgattttgagt
tttgtgtgtgattgcttttttcacaatcagtgttttcaggattatgtgatg
aactagatcttcaagtttcgttacatttcatatgttttcggaactcacga
agtacatattgggtattgtgctcaaaaaattcagcaatcagcttcgctcc
gctgactttagaacccaaaaaaatagtatggccaaactgactgtgttacg
atcatttcaatttttcaatacatatttaagatttctaagagtaagaaggt
caaaaactgttctggaatacatatatatttttcaggttacaattagtcaa
aaagtgcactgaaatatacgtttttaatttcacgaataacccaattagttc
aatgtattttttggtcaaccaacgttaaagtttggcttccaaccaattatc
atttctgatcaaccacaatgtttttttctttatctgcaagttaatttttta
tttttatccagatgtttggcatatttttcaattcttcactagcgcccact
tcttgcacttccggcgcccctgaatctaatgcatctgttgcaagaattgaa
agaccaatcaacacattgttttcttcacgagatactgaagaaaatgaata
aaaacagagaaaaagagccatgtgattagtgacaactgttgctaacagat
accatagcttggacttggtacgtgatggcaacgtatgggtcaacaaaaat
gattgcagagggggtgcaaaacagtcaagtcgagaaaatatgaaaaacag
aaaacaaagaacagaaaaatgggtttgagagtcagtataatttataaaag
aaaaattgtacatagaaattaaccattttttgtagaagaagttattttttca
agcatcgttaaaaattattcaaagcacctttatttcatatttaatttttaaa
catggttaaatgaacaacacggtgcgcaatcaggaaaacttgaaatctga
aactgttgttgtgatcttcttcgcaactgttcagatagcactagtgtaat
gttaagagtgcgcgaatataatggaatataatggatcacacctcctgcca
tcaggtaaacgtctctgttatcacatatttccaactattaaatttttacc
ttttacagttttacattttttttgaaaaaagtaactttttttgtcttcaaaat
ccctgacgaaaatatcaaatatttttaatcgagactgcagaggaaccgatt
gatgatttggaaaatccagctttacctgtgtaagaactgaaaagtttcat
aaccctagggtattcccagttacattccccactggctaacaatagcaccc
agtttttcatcacctttcttcaaatttctcggcgatttgttaaaacaaa
atttgtgtcccttctctgatatctctatgtctctaaacacaagttcatcg
gaaaacgaaggagggtaggtgttggttgggctcccgaagtgaaaatagaa
gagcaagaatagaatattggagagagagtgcagagagggcgggatagctc
ccgggattccgtttttcttcttcttctttatcttcaacgatgatgtgtgtgcgt
gttgtatagattctgttgctcccccacaactcgctccgaaggctcaatac
aattcaattgatattggaggagagcctaccggagtgggaggataagaaga
aacataagaagaagaagaagaagaagcatgcttctggtttttgatgctat
gaaaacggcacaaaaagatgattgaggtcccttttcaatacctttctctca
tctttcaaatcccattgaaacctaaaacttctcaccacgctttaccattg
ttctccaaaaacttatagcaatgtctataactttttttatctctgaaaagc
agtgttccatttttcttttttcctatttatttcaattgtttctcacattt
cgtttggattctttgcttgtcaaccagcttcttcttccacttttaccgtc
taattttcag

Synthetic 5' UTR *XbaI*
GGCAGGGAGCCATCAAACCCACGACCACTAGATCCA**TCTA**
ClaI *NotI*
GAGGATCCT**ATCGATTCGCGGCCGC**TGTACACCCGGGTGCCCTATAGTGA
KpnI *AgeI*
GTCGTATT**GGTACCGGT**AGAAAAA

***gfp* Exon 1** ATGAGTAAAGGAGAAGAACTTTTCAC
TGGAGTTGTCCCAATTCTTGTTGAATTAGATGGTGATGTTAATGGGCACA
AATTTTCTGTCAGTGGAGAGGGTGAAGGTGATGCAACATACGGAAAACTT
ACCCTTAAATTTATTTGCACTACTGGAAAACTACCTGTTCCATGG

Synthetic gfp intron 1 gtaag
tttaaacatatatatactaactaaccctgattatttaaattttcag

***gfp* Exon 2 (Ser65->Cys)** CCAA
CACTTGTCACTACTTTCTgTTATGGTGTTCAATGCTTcTCGAGATACCCA
GATCATATGAAACAGCATGACTTTTTCAAGAGTGCCATGCCCGAAGGTTA
TGTACAGGAAAGAACTATATTTTTCAAAGATGACGGGAACTACAAGACAC

Synthetic gfp intron 2
gtaagtttaaacagttcggtactaactaaccatacatatttaaattttca
g

***gfp* Exon 3**
GTGCTGAAGTCAAGTTTGAAGGTGATACCCTTGTTAATAGAATCGAGTT
AAAAGGTATTGATTTTAAAGAAGATGGAAACATTCTTGGACACAAATTGG
AATACAACTATAACTCACACAATGTATACATCATGGCAGACAAACAAAAG
AATGGAATCAAAGTT

Synthetic gfp intron 3
gtaagtttaaacatgatttactaactaactaatc
tgatttaaattttcag

***gfp* Exon 4**
AACTTCAAAATTAGACACAACATTGAAGATGGAA
GCGTTCAACTAGCAGACCATTATCAACAAAATACTCCAATTGGCGATGGC
CCTGTCCTTTTACCAGACAACCATTACCTGTCCACACAATCTGCCCTTTC
GAAAGATCCCAACGAAAAGAGAGACCACATGGTCCTTCTTGAGTTTGTAA
CAGCTGCTGGGATTACACATGGCATGGA

Synthetic GFP/DAM Linker cGAACTATACAAAagcccacaA
GCTAATTCGCTAGCCGGCCATACAAGTAATccggtcGAACAGAAACTCAT
CTCTGAAGAGGATCTGGCACCCGGC

E. coli *dam* (exon 1) AAGAAAAATCGCGCTTTTTTGAAGT
GGGCAGGGGGCAAGTATCCCCTGCTTGATGATATTAAACGGCATTTGCCC
AAGGGCGAATGTCTGGTTGAGCCTTTTGTAGGTGCCGGGTCGGTGTTTCT
CAACACCGACTTTTCTCGTTATATCCTTGCCGATATCAATAGCGACCTGA
TCAGTCTCTATAACATTGTGAAGATGCGTACTGATGAGTAC

Synthetic *dam* intron 1 gtaagttta
aacgatttcatactaactaacttcttgaatttaaattttcag

E. coli *dam* (exon 2) GTACAGGC
CGCACGCGAGCTGTTTGTTCCCGAAACAAATTGCGCCGAGGTTTACTATC
AGTTCCGCGAAGAGTTCAACAAAAGCCAGGATCCGTTCCGTCGGGCGGTA
CTGTTTTTATATTTGAACCGCTACGGTTACAACGGCCTGTGTCGTTACAA
TCTGCGCGGTGAGTTTAACGTGCCGTTCGGCCGCTACAAAAAACCCTATT
TCCCGGAAGCAGAGTTGTATCACTTCGCTGAAAAAGCGCAGAATGCCTTT
TTCTATTGTGAGTCTTACGCCGATAGCATGGCGCGCGCAGATGATGCATC
CGTCGTCTATTGCGATCCGCCTTATGCACCGCTGTCTGCGACCGCCAACT
TTACGGCGTATCACACAAACAGTTTTACGCTTGAACAACAAGCGCATCTG
GCGGAGATCGCCGAAGGTCTGGTTGAGCGCCATATTCCAGTGCTGATCTC
CAATCACGATACGATGTT

Synthetic *dam* intron 2
gtaagtttaaacaatagtttactaactaacct
ttcagatttaaattttcag

E. coli *dam* (exon 3)
AACGCGTGAGTGGTATCAGCGCGCAAAATTG
CATGTCGTCAAAGTTCGACGCAGTATAAGCAGCAACGGCGGCACACGTAA
AAAGGTGGACGAACTGCTGGCTTTGTACAAACCAGGAGTCGTTTCACCCG
CGAAAAAATAG

EcoRI **Synthetic Linker**
tcttctgctagccggc**GAATTC**CAACTGAGCGCCGGTCG
CTACCATTACCAACTTGTCTGGTGTCAAAAATAATAGGGGCCGCTGTCAT
CAGAgtaagtttaaactgagttctactaacgagtaatatttaaatt
ttcag

unc-54 3' UTR
CATCTCGCGCCCGTGCCTCTGACTTCTAAGTCCAATTACTCTTCA
ACATCCCTACATGCTCTTTCTCCCTGTGCTCCCACCCCCTATTTTTGTTA
TTATCAAAAAAACTTCTTCTTAATTTCTTTGTTTTTTAGCTTCTTTTAAG
TCACCTCTAACAATGAAATTGTGTAGATTCAAAAATAGAATTAATTCGTA
ATAAAAAGTCGAAAAAAATTGTGCTCCCTCCCCCCATTAATAATAATTCT
ATCCCAAAATCTACACAATGTTCTGTGTACACTTCTTATGTTTTTTTTAC
TTCTGATAAATTTTTTTTG**AAA**

unc-54 3' Flank catcatagaaaaaaccgcacacaaaata
ccttatcatatgttacgtttcagtttatgaccgcaatttttatttcttcg
cacgtctgggcctctcatgacgtcaaatcatgctcatcgtgaaaaagttt
tggagtatttttggaatttttcaatcaagtgaaagtttatgaaattaatt
ttcctgcttttgctttttgggggtttcccctattgtttgtcaagagtttc
gaggacggcgtttttcttgctaaaatcacaagtattgatgagcacgatgc
aagaaagatcggaagaaggtttgggtttgaggctcagtggaaggtgagta
gaagttgataatttgaaagtggagtagtgtctatggggtttttgccttaa
atgacagaatacattcccaatataccaaacataactgttt

SplI *ApaI* **Plasmid Vector Backbone** cctactagtc
ggc**cgtacgggccc**tttcgtctcgcgcgtttcggtgatgacggtgaaaac
ctctgacacatgcagctcccggagacggtcacagcttgtctgtaagcgga
tgccgggagcagacaagcccgtcagggcgcgtcagcgggtgttggcgggt
gtcggggctggcttaactatgcggcatcagagcagattgtactgagagtg
caccatatgcggtgtgaaataccgcacagatgcgtaaggagaaaataccg
catcaggcggccttaagggcctcgtgatacgcctatttttataggttaat
gtcatgataataatggtttcttagacgtcaggtggcacttttcggggaaa
tgtgcgcggaacccctatttgtttatttttctaaatacattcaaatatgt
atccgctcatgagacaataaccctgataaatgcttcaataatattgaaaa
aggaagagtatgagtattcaacatttccgtgtcgcccttattcccttttt
tgcggcattttgccttcctgtttttgctcacccagaaacgctggtgaaag
taaaagatgctgaagatcagttgggtgcacgagtgggttacatcgaactg
gatctcaacagcggtaagatccttgagagttttcgccccgaagaacgttt
tccaatgatgagcacttttaaagttctgctatgtggcgcggtattatccc
gtattgacgccgggcaagagcaactcggtcgccgcatacactattctcag
aatgacttggttgagtactcaccagtcacagaaaagcatcttacggatgg
catgacagtaagagaattatgcagtgctgccataaccatgagtgataaca
ctgcggccaacttacttctgacaacgatcggaggaccgaaggagctaacc
gcttttttgcacaacatgggggatcatgtaactcgccttgatcgttggga
accggagctgaatgaagccataccaaacgacgagcgtgacaccacgatgc
ctgtagcaatggcaacaacgttgcgcaaactattaactggcgaactactt
actctagcttcccggcaacaattaatagactggatggaggcggataaagt
tgcaggaccacttctgcgctcggcccttccggctggctggtttattgctg
ataaatctggagccggtgagcgtgggtctcgcggtatcattgcagcactg
gggccagatggtaagccctcccgtatcgtagttatctacacgacggggag
tcaggcaactatggatgaacgaaatagacagatcgctgagataggtgcct
cactgattaagcattggtaactgtcagaccaagtttactcatatatactt
tagattgatttaaaacttcatttttaatttaaaaggatctaggtgaagat
cctttttgataatctcatgaccaaaatcccttaacgtgagttttcgttcc
actgagcgtcagaccccgtagaaaagatcaaaggatcttcttgagatcct
ttttttctgcgcgtaatctgctgcttgcaaacaaaaaaaccaccgctacc
agcggtggtttgtttgccggatcaagagctaccaactctttttccgaagg
taactggcttcagcagagcgcagataccaaatactgtccttctagtgtag
ccgtagttaggccaccacttcaagaactctgtagcaccgcctacatacct
cgctctgctaatcctgttaccagtggctgctgccagtggcgataagtcgt
gtcttaccgggttggactcaagacgatagttaccggataaggcgcagcgg
tcgggctgaacggggggttcgtgcacacagcccagcttggagcgaacgac
ctacaccgaactgagatacctacagcgtgagcattgagaaagcgccacgc
ttcccgaagggagaaaggcggacaggtatccggtaagcggcagggtcgga
acaggagagcgcacgagggagcttccagggggaaacgcctggtatcttta
tagtcctgtcgggtttcgccacctctgacttgagcgtcgatttttgtgat
gctcgtcaggggggcggagcctatggaaaaacgccagcaacgcggccttt
ttacggttcctggccttttgctggccttttgctcacatgttctttcctgc
gttatcccctgattctgtggataaccgtattaccgcctttgagtgagctg
ataccgctcgccgcagccgaacgaccgagcgcagcgagtcagtgagcgag
gaagcggaagagcgcccaatacgcaaaccgcctctccccgcgcgttggcc
gattcattaatgcagctggcacgacaggtttcccgactggaaagcgggca
gtgagcgcaacgcaattaatgtgagttagctcactcattaggcaccccag
gctttacactttatgcttccggctcgtatgttgtgtggaattgtgagcgg
ataacaatttcacacaggaaacagctatgaccatgattacgccaagctgt
aagtttaaacatgatcttactaactaactattctcatttaaattttcaga
gcttaaaaatggctgaaatcactcacaacgatggatacgctaacaacttg
gaaatgaaat
