# Supplementary material for: Distributed probing of chromatin structure in vivo reveals pervasive chromatin accessibility for expressed and non-expressed genes during tissue differentiation in C. elegans
Source: BMC Genomics. 2010 Aug 6;11:465. doi: 10.1186/1471-2164-11-465 (PMC3091661; doi:10.1186/1471-2164-11-465)
Supplement: Additional file 2 — Supplemental materials, including notes on methods and supplemental figures and tables in Microsoft Word format. [file 1471-2164-11-465-S2.PDF]

ADDITIONAL FILE 2

Distributed probing of chromatin structure *in vivo* reveals pervasive chromatin accessibility for expressed and non-expressed genes during tissue differentiation in *C. elegans*

Ky Sha[1,2,3], Sam G Gu[1], Luiz C Pantalena-Filho[2,3], Amy Goh[2,3], Jamie Fleenor[2], Daniel Blanchard[1,2,3], Chaya Krishna[1], & Andrew Fire[1,2,3,§]

[1]Depts. of Pathology and Genetics, Stanford University School of Medicine, 300 Pasteur Drive, Palo Alto CA, USA

[2]Carnegie Institution of Washington, 115 West University Parkway, Baltimore MD, USA

[3]Biology Department, Johns Hopkins University, 3400 North Charles St., Baltimore MD, USA

[§]Corresponding author: Andrew Fire (afire@stanford.edu)

SUPPLEMENTAL METHOD

Additional Notes on DALEC

It is necessary to strictly limit the region of complementarity between the top and bottom strands of Linker A (Figure 3, inset) to at most 19bp. Because Linker A–Linker A dimers can form at Step 2 of the protocol, complementarity of 19bp or greater would allow *Mme I* to cut at the double-stranded region of its partner linker, thereby allowing the restricted fragment to be cloned as an insert and reducing the number of Linker A molecules in the process. The extent to which Linker A–Linker A dimers are susceptible to *Mme I* restriction may be sequence-dependent. The majority of sequences with 19bp or less are resistant to *Mme I* restriction. However, for the sequence GCCTCCCTCGCGCCATCAG$(N)_{12}$TCCTCATTCTCTCCGAC, we have found that greater than 50% of the amplicons contained linker sequence as the insert, even when the double-stranded region (underlined) is only 17bp. Hence, it is necessary to confirm the integrity of the library with Sanger sequencing before the high throughput sequencing step.

SUPPLEMENTAL FIGURES

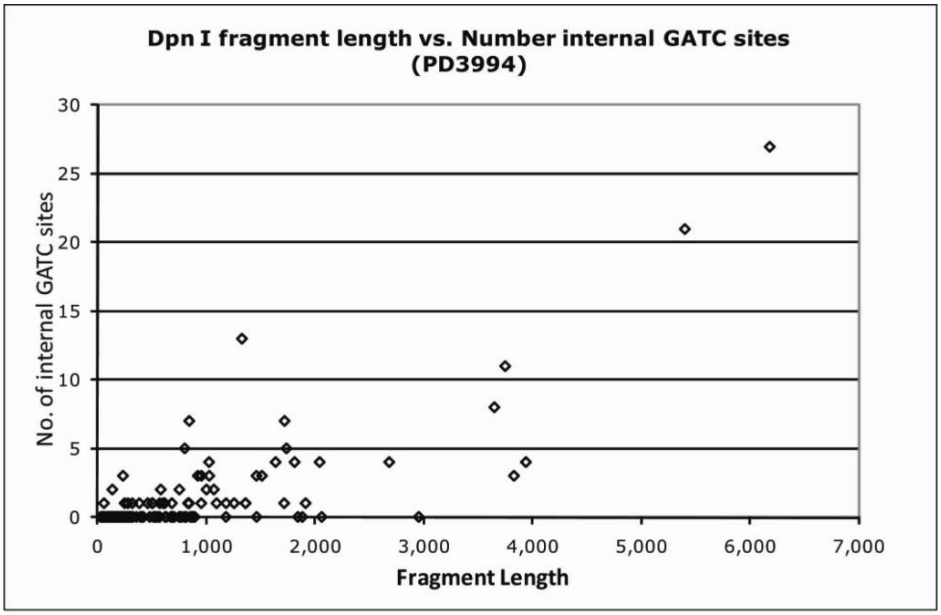

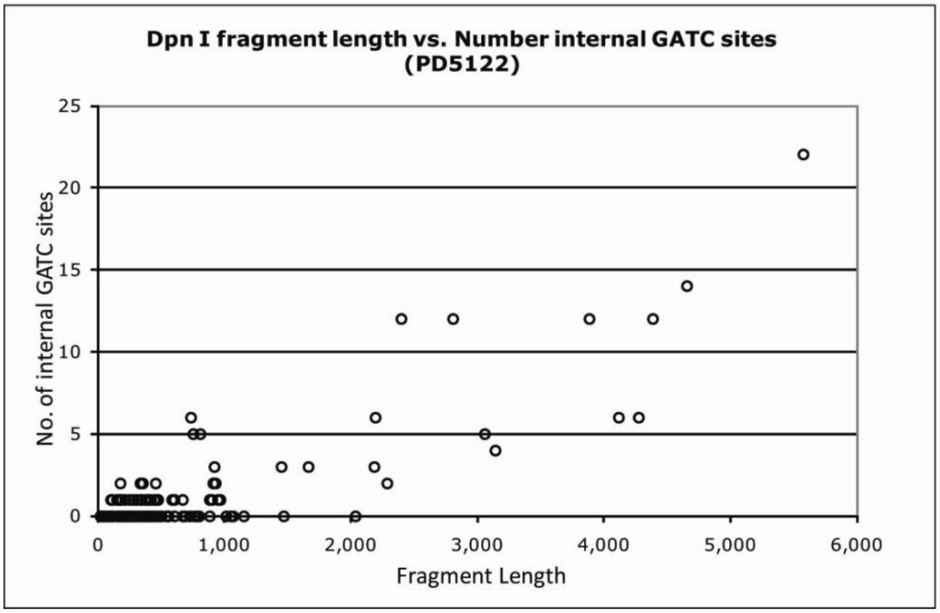

Figure S2. ***Dpn I*** **fragment length versus number of uncut internal (i.e. non-methylated) GATC sites in each fragment.**

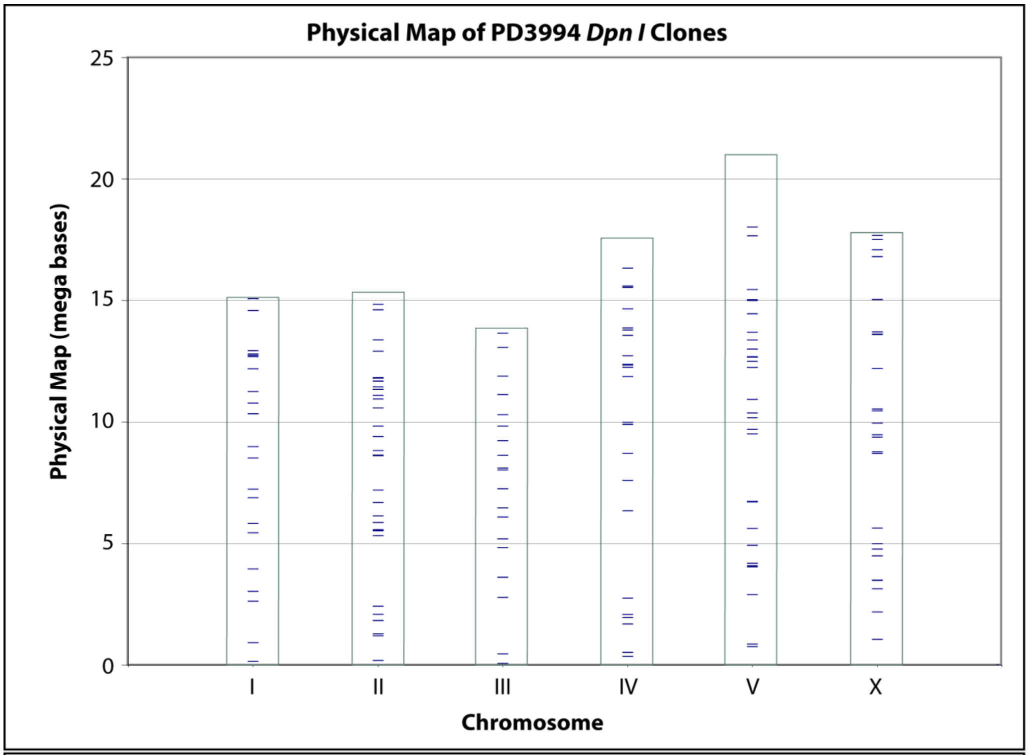

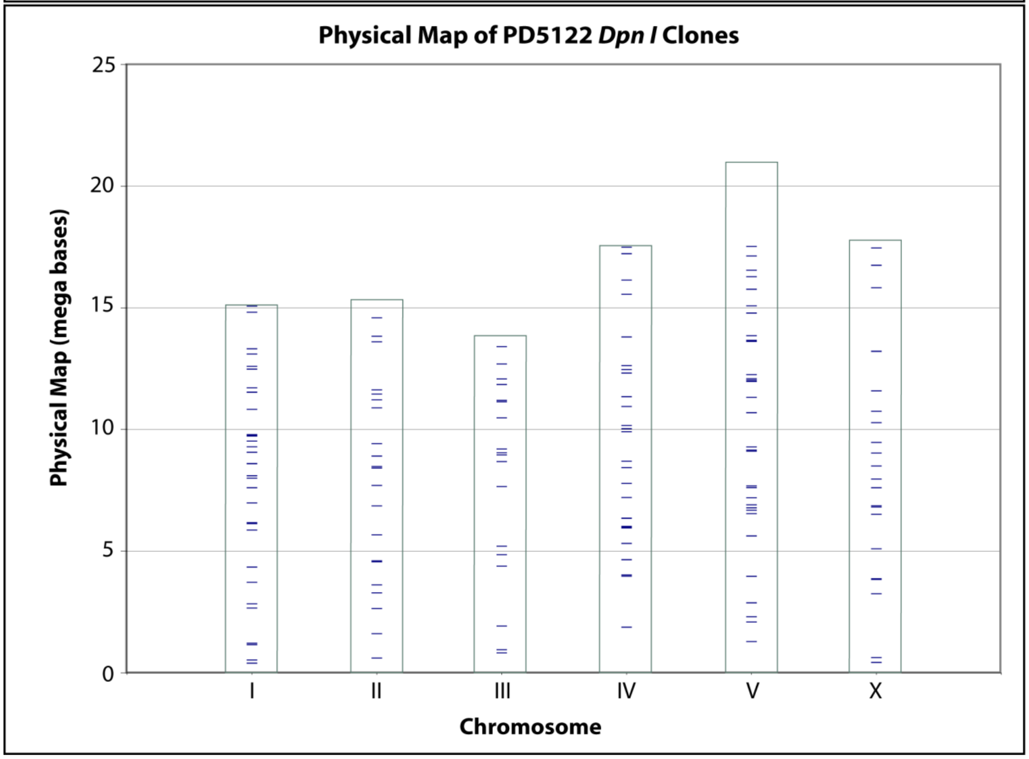

Figure S3. Genomic distribution of captured *Dpn I* fragments. Each column represents a chromosome, scaled to the appropriate physical size.

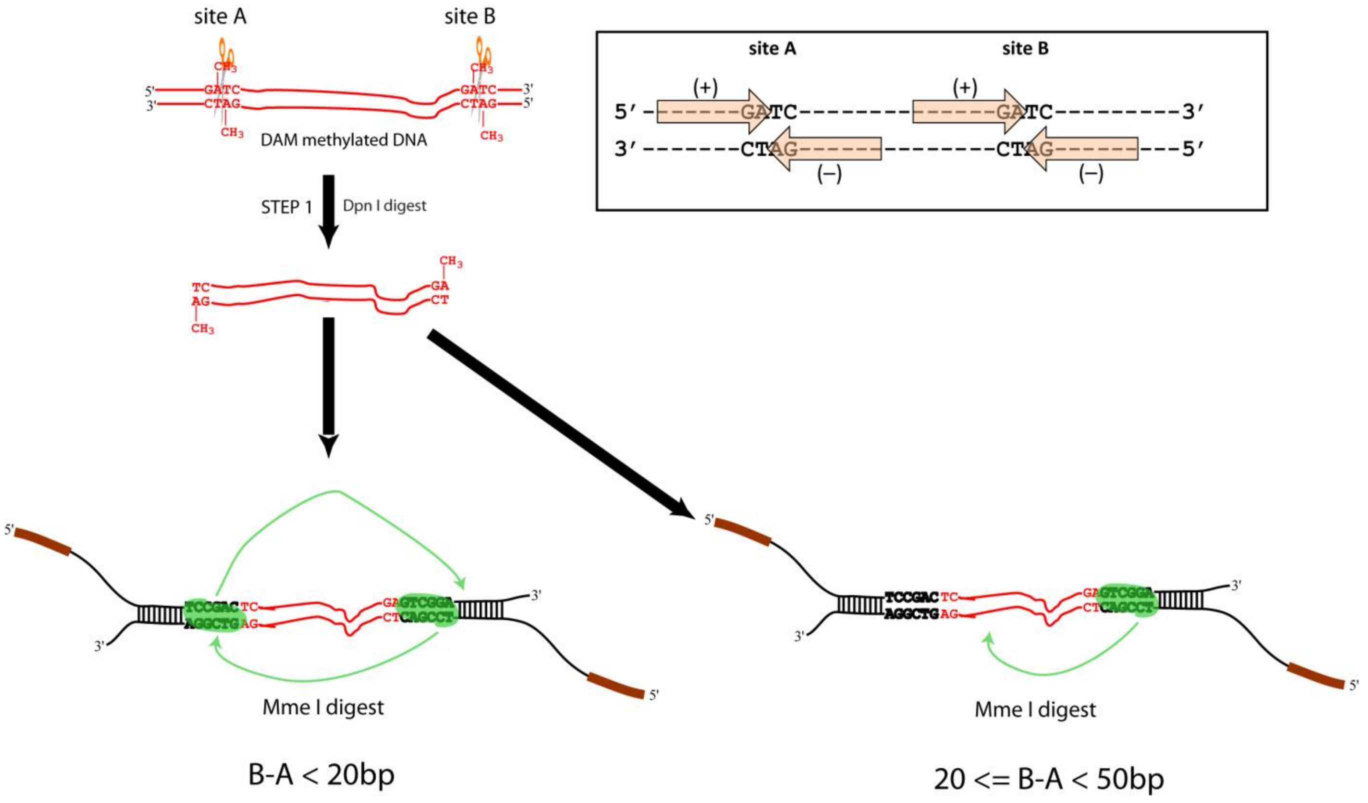

Figure S4. Filtering of "proximal tags". Under the assumption that every GATC site in genomic (naked) DNA has the same probability of being methylated, then in theory any two adjacent GATC sites A and B will yield four Dam tags (inset, pink arrows). When the distance separating A and B is greater than 50bp, the frequency with which each tag can be captured by DALEC should be independent of each other. However, we have observed that when A and B are less than 50bp apart, the events are no longer independent. There are two scenarios that account for this. First, when the separation distance is less than 20bp, DALEC will always capture one tag at the expense of the other tag. This is because *Mme I* will cut into the opposite Linker A. The resulting captured tag will thus be part genomic sequence and part linker sequence. Under this scenario, we excluded from analysis all four tags associated with two adjacent GATC sites less than 20bp apart. Second, when the distance between two adjacent sites is equal to or greater than 20bp but less than 50bp, the minus tag of site A [A(–)] and plus tag of site B [B(+)] get eliminated. Exclusion of the inner two tags is necessary because any sequence-bias of *Mme I* binding would lead to preferential capture of one of the (inner) tags at the expense of the other. Although proximal tags constitute ≈23% of all potential Dam tags, we saw minimal differences in our analysis results using the filtered versus unfiltered *in silico* data set.

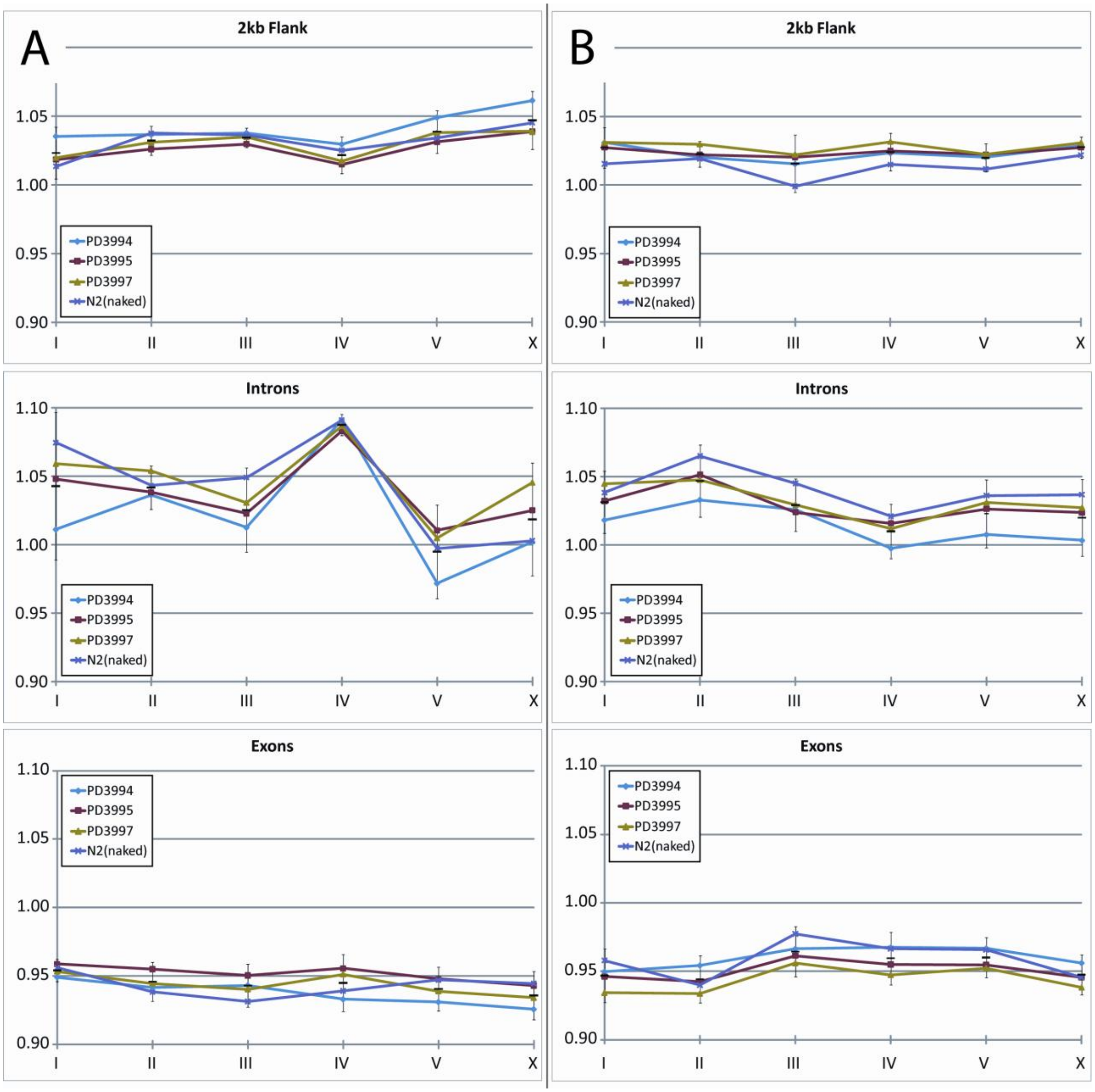

Figure S5. Minimal differences in promoter-specific DAM accessibility across genomic modalities. Panel A refers to the set of 3,904 genes analyzed in Figure 6 of the article. Panel B contains all other annotated *C. elegans* genes, excluding those found in Panel A. For a given gene, the DAM accessibility index was calculated for its exonic, intronic, and the 2kb flanking region (2kb upstream plus downstream of the gene boundary). We defined the accessibility index (vertical axis) to be the number of tags per GATC site in that feature (exon, introns, or flanking), normalized over the number of tags per GATC site for the entire chromosome. The horizontal axis represents chromosomes. Error bars represent two standard deviations from the mean.

SUPPLEMENTAL TABLES

Table S1. PD3994 Cloning Summary

			I	II	III	IV	V	X	M	Total
		total clones:	26	34	29	26	31	33	3	182
unique clones		unique clones:	23	32	24	25	30	31	3	168
unique clones	*Dpn I* clones	confirmed Dpn I clones:	22	31	20	24	27	26	3	153
unique clones	*Dpn I* clones	size range:	39-6,181	31-1,330	52-1,885	33-3,650	49-5,401	35-3,940	189-1,465	na
unique clones	*Dpn I* clones	avg. length of fragment:	971	432	470	742	1000	880	614	730
unique clones	*Dpn I* clones	avg. internal GATC per frag:	1.9	0.7	0.5	0.9	2.2	1.1	0.0	1.2

Table S1. PD5122 Cloning Summary

			I	II	III	IV	V	X	M	Total
		total clones:	34	22	22	32	35	25	4	174
unique clones		unique clones:	33	21	20	32	34	23	4	167
unique clones	*Dpn I* clones	confirmed Dpn I clones:	33	21	18	29	34	23	3	161
unique clones	*Dpn I* clones	size range:	43-4,662	42-2,187	25-5,581	40-2,294	51-4,117	26-4,388	48-308	na
unique clones	*Dpn I* clones	avg. length of fragment:	635	462	699	466	701	863	205	576
unique clones	*Dpn I* clones	avg. internal GATC per frag:	1.2	0.6	2.4	0.6	1.1	1.2	0.3	1.1

Table S1. Summary of cloned *Dpn I* fragments from lines PD3994 and PD5122. "Unique clones" indicate non-redundant fragments. "Confirmed *Dpn I* clones" refer to fragments confirmed to have been flanked by GATC sites at both ends (i.e. generated by *Dpn I* restriction and not by spurious DNA fragmentation).

Table S2. PD3994 *Dpn I* Fragment Hits

	gene	gene score	span	strand	physical map	frag length	internal GATC	repetitive?
Chromosome I	C32F10.6(flanking)	1	na	-	5,826,419	397	0	no
	F27C1.11	1	exon/intron	-	5,439,301	676	0	no
	F31C3.10	1	exon	-	15,068,878	191	0	yes
	F31C3.9	1	exon	+	15,073,791	39	0	no
	F32H2.5	1	exon/intron	-	8,984,085	178	0	no
	F46A8.3	1	exon/intron	+	11,250,522	1,464	3	yes
	F47B3.8	1	exon	+	3,947,018	329	0	no
	F56C11.1	1	intron	-	148,515	308	0	no
	K02B12.3	1	exon/intron	-	8,518,226	607	1	no
	R05D7.gc4(flanking)	1	exon/intron	+	12,182,659	3,830	3	no
	R06C7.2	1	exon/intron	+	7,238,349	6,181	27	no
	R13H8.1	1	intron	+	10,776,534	812	0	no
	T21G5.2	1	exon	+	6,879,427	50	0	no
	T26E3.2	1	exon/intron	+	12,687,319	624	0	no
	W02A11.3	1	exon/intron	+	12,746,982	1,097	1	no
	Y105E8A.24	1	exon	+	14,584,230	1,739	5	no
	Y18D10A.1	1	intron	-	12,797,076	752	0	yes
	Y18D10A.21	1	exon	-	12,937,684	136	0	no
	Y23H5A.3	1	exon/intron	-	2,620,506	844	1	no
	Y54E10BR.5	0.5	exon	+	3,031,057	170	0	no
	Y95B8A.12	1	intron	-	917,465	54	0	no
	ZC434.3	0.5	exon	+	10,343,592	881	0	no
	ZC434.5	0.5	exon	+	10,343,592	881	0	no
Chromosome II	B0491.5	1	exon/intron	-	11,344,558	504	1	no
	C09H10.3	1	exon/intron	+	11,099,337	552	0	no
	C14A4.1	1	exon/intron	+	10,580,284	151	0	no
	C17C3.2	0.5	exon/intron	-	5,568,071	322	1	no
	C29H12.1	1	exon/intron	+	6,132,141	1,072	2	no
	C47D12.2	1	exon	-	11,678,682	73	0	no
	E04F6.5	1	exon/intron	+	7,192,861	113	0	no
	F08B1.1	1	intron	+	5,325,620	776	0	no
	F19H8.4	1	exon	-	14,617,422	252	1	no
	F28C6.10	0.5	exon/intron	+	8,607,551	755	2	no
	F28C6.8	0.5	exon	+	8,607,551	755	2	no
	F28C6.9	1	exon/intron	+	8,610,238	149	0	no
	F42G2.4	1	intron	+	2,413,484	237	0	no
	F43G6.6	1	exon/intron	-	11,806,795	533	0	no
	F46C5.3(flanking)	1	na	-	8,825,296	187	0	no
	F53A10.2(flanking)	1	na	+	1,196,229	256	0	no
	F58G1.2	1	exon	+	12,917,389	302	0	no
	H43E16.1	1	exon/intron	+	6,678,363	835	1	no
	R03D7.7	1	exon	-	10,952,941	35	0	no
	R05H10.6	1	exon/intron	+	14,843,772	1,184	1	no
	T08E11.4	1	exon/intron	-	1,829,108	868	0	yes
	T16A1.7	1	exon	+	2,085,547	204	0	yes
	T22C8.7	1	exon/intron	-	8,632,651	686	0	no
	W02B12.1	1	exon/intron	+	11,449,394	628	0	no
	Y48C3A.12	1	intron	-	13,379,884	550	0	no
	Y51B9A.7	1	exon	-	9,400,307	31	0	no
	Y57G7A.12	1	intron	+	1,279,406	314	0	yes
	Y6D1A.1	1	exon/intron	+	11,821,009	1,330	13	yes
	ZK177.10(flanking)	1	na	+	5,524,406	184	0	no
	ZK938.1	1	exon/intron	+	9,830,288	204	0	no
Chromosome III	C05D10.4	1	exon/intron	+	6,089,418	92	0	no
	C13G5.1	1	exon/intron	+	8,622,968	425	0	no
	C14B9.4	0.5	exon	-	8,103,623	846	7	no
	C48D5.2	1	exon/intron	-	3,608,360	763	0	no
	F26A1.10	0.5	exon	+	4,830,227	128	0	no
	F44B9.7	1	exon/intron	+	8,025,228	524	0	no
	K10D2.1	1	exon/intron	+	5,189,126	897	0	no
	K11H3.4	1	exon/intron	+	9,833,059	417	0	no
	T07A5.1	1	exon/intron	+	10,304,789	1,885	0	no
	T12D8.1	1	exon	-	13,649,071	233	0	no
	T23G5.1	1	exon	-	9,228,026	52	0	no
	T26A5.9	1	exon/intron	+	6,463,396	283	1	no
	W05G11.6	0.5	exon	-	62,756	279	0	no
	Y39E4B.6	1	exon/intron	+	13,070,560	387	1	no
	Y48A6C.5	0.5	exon	-	11,135,542	575	0	yes
	Y55B1BR.5	1	exon/intron	-	455,112	150	0	no
	Y56A3A.5(flanking)	1	na	+	11,886,224	99	0	no
	Y71H2AM.19	1	exon	-	2,778,878	160	0	no

Tables S2-S3. Annotated *C. elegans* sequences that correspond to captured *Dpn I* fragments. The “gene score” column is an arbitrary scoring scheme designed to “weigh” each gene as a function of its position to the nearest *Dpn I* fragment. The algorithm was as follows: (1) if a *Dpn I* fragment completely spanned a gene or if the fragment completely lied within the gene, the gene was given a score of 1.0. (2) If a gene spanned the *Dpn I* fragment on only one side, that gene was given 0.5 points. In cases where the fragment spanned two genes, the 0.5 score was given to the gene with the greater overlap. (3) If a *Dpn I* fragment mapped within 1kb upstream of a gene’s start site, it was given a score of 1.0. “na” indicates the nearest gene was outside of the 1kb limit on either side of the fragment.

Table S2(continued)

Table S2 (*cont.*) PD3994 *Dpn I* Fragment Hits

	gene	gene score	span	strand	physical map	frag length	internal GATC	repetitive?
Chromosome IV	B0513.5	1	exon/intron	-	13,872,928	804	0	no
	C29E6.2	1	exon	+	11,866,026	207	0	no
	C35D6.10	0.5	exon	+	16,332,680	321	0	no
	C47E12.6	1	exon/intron	+	9,987,900	322	0	no
	F19B6.4(flanking)	1	na	+	12,341,479	1,639	4	no
	F28D1.1	1	exon/intron	-	12,372,687	881	0	no
	F32B6.8(flanking)	1	na	+	9,898,347	361	0	no
	F52C12.2	1	exon	-	1,955,422	33	0	no
	H23L24.5	1	exon/intron	-	8,714,785	226	0	no
	R05C11.3	0.5	exon/intron	+	2,080,669	3,650	8	no
	T23F6.3	0.5	exon/intron	+	12,724,341	2,044	4	no
	T23F6.4	0.5	exon/intron	+	12,724,341	2,044	4	no
	W03G1.6	1	intron	-	512,775	819	0	no
	Y105C5A.4	1	exon/intron	+	15,589,800	2,063	0	yes
	Y2C2A.1	1	exon	-	7,595,176	139	2	yes
	Y45F10B.10	1	intron	+	13,565,861	46	0	no
	Y45F10D.3	1	exon/intron	+	13,779,573	292	0	no
	Y54G2A.12	0	exon	+	2,782,764	921	1	no
	Y57G11C.33	1	exon/intron	+	14,657,230	574	1	no
	Y66H1B.1	1	intron	+	353,442	527	0	yes
	Y73B6BL.15	1	exon	-	6,345,446	193	0	yes
	Y73F8A.35	1	exon/intron	+	15,537,191	956	1	no
	Y94H6A.1	1	exon/intron	+	2,745,268	220	0	no
Chromosome V	C02E7.5	0.5	exon/intron	+	4,920,424	958	3	yes
	C02E7.8	0.5	exon/intron	+	4,920,424	958	3	yes
	C05E4.3	1	exon	+	754,701	49	0	no
	C31B8.gc1	1	exon	+	2,895,397	70	0	no
	C49G7.7	1	exon/intron	-	4,037,163	405	0	no
	F11A3.1	0.5	exon/intron	+	9,512,616	1,722	7	no
	F11A3.3	0.5	exon/intron	+	9,512,616	1,722	7	no
	F14H8.6	1	exon/intron	-	15,033,898	233	0	no
	F15B9.1	1	exon/intron	+	12,999,329	1,030	3	no
	F23B12.6	1	exon/intron	-	14,453,905	5,401	21	no
	F58B4.6	0.5	exon	-	10,927,841	180	0	no
	F59A1.11	1	exon/intron	+	17,664,183	1,362	1	no
	K07C5.8	1	exon/intron	-	10,365,083	138	0	no
	K09C6.2(flanking)	1	na	-	855,933	530	0	no
	M03E7.2	1	exon/intron	-	5,620,508	270	0	no
	M04G12.1	1	intron	+	13,376,858	3,749	11	no
	T01C3.5	1	exon/intron	+	14,999,764	868	0	no
	T01D3.1	1	exon/intron	+	13,695,815	2,955	0	no
	T04H1.2	1	exon/intron	-	12,246,499	619	1	no
	T23F1.1	0.5	exon/intron	+	15,453,880	1,718	1	no
	T26H5.5	0.5	exon	+	15,453,880	1,718	1	no
	W02F12.3	0.5	exon/intron	+	6,706,827	584	2	no
	W02F12.4	0.5	exon/intron	+	6,706,827	584	2	no
	W04D2.3	0.5	exon	+	12,494,990	1,028	4	no
	W04D2.5	0.5	exon	+	12,494,990	1,028	4	no
	Y45G5AM.1	1	exon	+	4,187,809	237	3	no
	Y59A8B.4	1	exon	-	18,017,937	178	0	yes
	ZC487.1	1	exon/intron	+	6,730,996	158	0	no
	ZK287.8	1	intron	-	9,701,877	464	1	no
Chromosome X	C08A9.5	1	exon/intron	+	17,088,412	3,940	4	no
	C17G1.6	1	exon/intron	-	9,947,229	92	0	no
	C18B12.5	1	exon	-	15,043,089	103	0	no
	C39B10.1	1	exon/intron	-	10,460,519	925	3	no
	C39E6.6	1	exon/intron	+	4,767,265	510	1	no
	E01H11.1	1	intron	+	9,376,512	59	0	no
	F41G4.2	1	exon/intron	+	16,808,264	571	0	no
	F48B9.1	1	exon	-	2,180,977	106	0	no
	F53A9.3	0.5	exon/intron	+	8,707,025	1,514	3	no
	F53A9.4	0.5	exon	+	8,707,025	1,514	3	no
	F54B11.6	0.5	exon/intron	+	13,597,226	1,915	1	no
	K02D3.1(flanking)	1	na	+	13,705,538	1,183	0	no
	K03E6.3(flanking)	1	na	+	1,051,518	2,684	4	no
	K10B3.1	1	exon/intron	-	3,135,727	856	0	no
	M02D8.1(flanking)	1	na	+	8,763,355	174	0	no
	T23E7.2	1	exon/intron	-	17,674,064	635	0	no
	ZC8.6	1	exon/intron	+	4,992,155	1,814	4	no
	ZK899.8	1	intron	+	9,472,807	190	0	no
mito	MTCE.25	1	exon	+	7,439	189	0	no
	MTCE.34	1	exon	+	10,778	1,465	0	no

Table S3

Table S3. PD5122 *Dpn I* Fragment Hits

chromosome	gene	gene score	span	strand	physical map	frag length	internal GATC	repetitive?
Chromosome I	C16C2.3	1	intron	+	9,729,732	108	0	no
Chromosome I	C30F12.3	0.5	exon/intron	+	6,978,309	604	1	no
Chromosome I	C35E7.6	1	exon	-	10,825,487	224	0	no
Chromosome I	C41G7.1	0.5	exon	+	9,519,063	296	0	no
Chromosome I	D2030.8	1	exon/intron	+	7,601,919	351	0	no
Chromosome I	F14B4.2(flanking)	1	na	-	9,286,913	168	0	no
Chromosome I	F18C12.2	1	exon	+	8,095,918	349	1	no
Chromosome I	F26E4.10	1	exon	+	9,790,019	108	1	no
Chromosome I	F30A10.10	0.5	exon	+	9,519,063	296	0	no
Chromosome I	F31C3.7(flanking)	1	na	-	15,072,590	121	0	no
Chromosome I	F32B4.4	1	exon	-	11,533,060	124	0	no
Chromosome I	F33D11.8(flanking)	1	na	+	5,863,872	1,053	0	no
Chromosome I	F33E2.7(flanking)	1	na	+	12,594,484	161	0	no
Chromosome I	F40E.5(flanking)	1	na	+	2,657,784	215	0	no
Chromosome I	H05L14.2	1	exon/intron	+	7,996,507	4,662	14	no
Chromosome I	K02A11.1	1	exon/intron	+	9,748,866	972	1	no
Chromosome I	R05D11.3	1	exon/intron	+	8,596,243	4,275	6	no
Chromosome I	R119.5	1	intron	+	392,290	43	0	no
Chromosome I	T04D3.1(flanking)	1	na	+	13,312,849	400	0	no
Chromosome I	T09B4.6(flanking)	1	na	-	6,165,085	195	0	no
Chromosome I	T12F5.4	1	exon	-	3,716,015	184	2	no
Chromosome I	T22H2.6	1	intron	+	11,711,364	110	0	no
Chromosome I	T27A3.1	0.5	exon/intron	-	6,130,715	483	0	no
Chromosome I	T27F6.1(flanking)	1	na	-	12,477,338	106	1	yes
Chromosome I	W06D4.1	1	exon/intron	-	9,059,206	255	0	no
Chromosome I	Y26D4A.14	1	exon	+	13,102,883	216	0	no
Chromosome I	Y48G8AL.1	0.5	exon/intron	+	1,211,430	1,476	0	no
Chromosome I	Y48G8AL.10	1	intron	+	1,149,117	84	0	yes
Chromosome I	Y54E5B.gc1	1	intron	+	14,813,807	421	1	no
Chromosome I	Y65B4BL.6(flanking)	1	na	+	524,745	2,806	12	no
Chromosome I	ZK973.6	1	exon	-	4,343,548	150	1	yes
Chromosome II	B0457.1	1	exon	-	8,906,458	69	0	no
Chromosome II	B0495.5	1	exon	+	7,696,767	252	0	no
Chromosome II	C01G12.8(flanking)	1	na	+	14,592,588	550	0	no
Chromosome II	C04A2.7c(flanking)	1	na	-	6,858,598	318	0	no
Chromosome II	C08E3.3	1	exon	+	1,604,697	66	0	no
Chromosome II	C25H3.15	1	exon/intron	+	5,665,691	237	0	no
Chromosome II	F02E11.1	1	exon/intron	+	3,280,207	346	1	no
Chromosome II	F07A11.6	1	exon	+	11,625,701	48	0	no
Chromosome II	F08G2.10	0.5	exon	+	13,829,359	1,157	0	no
Chromosome II	F08G2.4	0.5	exon	+	13,829,359	1,157	0	no
Chromosome II	F22E5.9(flanking)	1	na	-	2,637,836	102	0	no
Chromosome II	F35D11.5	1	intron	+	4,602,051	383	0	no
Chromosome II	F44F4.1	1	exon/intron	-	10,882,684	343	1	no
Chromosome II	F49E12.6(flanking)	1	na	+	8,412,429	82	0	no
Chromosome II	M03A1.3	1	exon/intron	+	4,559,075	2,187	3	no
Chromosome II	M05D6.1	0.5	exon	-	8,475,138	317	0	no
Chromosome II	M176.1(flanking)	1	na	+	9,412,153	182	0	no
Chromosome II	T06D8.1	1	exon/intron	+	11,215,479	283	1	yes
Chromosome II	W02B12.3	1	intron	+	11,454,573	42	0	no
Chromosome II	Y48E1B.13	1	exon/intron	-	13,602,484	372	1	no
Chromosome II	Y49F6A.5	1	exon/intron	+	3,608,973	736	6	no
Chromosome III	B0284.1	1	exon	+	4,378,415	120	0	no
Chromosome III	B0412.2	1	exon	-	813,450	106	0	no
Chromosome III	B0523.5	1	exon	-	8,678,866	783	0	no
Chromosome III	B0524.1(flanking)	1	na	-	1,917,427	182	1	yes
Chromosome III	D2045.9	1	exon/intron	+	10,476,357	951	1	no
Chromosome III	F11F1.7	1	intron	-	13,404,918	113	0	no
Chromosome III	F26A1.14	0.5	exon	-	4,849,698	124	0	no
Chromosome III	K10D2.6	1	exon/intron	-	5,198,038	464	1	no
Chromosome III	R107.1	1	exon	-	9,039,603	108	1	no
Chromosome III	T12B5.11	1	exon/intron	+	948,039	2,404	12	yes
Chromosome III	Y111B2A.19	1	intron	-	12,690,230	464	2	no
Chromosome III	Y47D3A.6	1	intron	-	11,193,621	112	1	no
Chromosome III	Y48A6C.5	1	exon	-	11,135,441	101	0	no
Chromosome III	Y56A3A.1	1	exon	-	11,845,140	25	0	no
Chromosome III	Y75B8A.1	1	exon/intron	-	12,074,515	362	2	yes
Chromosome III	ZC84.6	1	exon/intron	+	9,197,546	158	0	no
Chromosome III	ZK643.3	0.5	exon/intron	+	8,949,523	5,581	22	no
Chromosome III	ZK643.5	0.5	exon/intron	+	8,949,523	5,581	22	no
Chromosome III	ZK783.4	0.5	na	-	7,649,899	431	0	no

Table S3 (continued)

Table S3 (*cont.*) PD5122 *Dpn I* Fragment Hits

	gene	gene score	span	strand	physical map	frag length	internal GATC	repetitive?
Chromosome IV	4R79.1	1	exon/intron	-	17,488,972	127	0	no
	B0350.2	1	exon	+	5,988,952	695	0	no
	C01C7.1	1	exon/intron	-	12,624,033	543	0	no
	C25G4.8	1	exon/intron	+	12,462,193	592	1	no
	C33H5.9	1	exon	+	7,779,848	350	0	no
	C46G7.2	1	exon/intron	-	6,016,626	909	1	no
	F08B4.1	1	exon/intron	-	8,694,239	914	2	no
	F29B9.8	1	exon/intron	+	4,648,221	215	0	no
	F32B6.8	0.5	exon	+	9,902,457	93	0	no
	F44D12.5	0.5	exon	+	10,029,078	754	5	no
	F55A8.2	1	intron	+	1,868,385	400	1	no
	K09E10.3(flanking)	1	na	+	12,319,016	190	1	yes
	R13A1.4	1	exon/intron	-	7,200,834	885	0	no
	T01G1.1	1	exon/intron	-	11,345,807	346	0	no
	T14G10.1	1	exon/intron	-	10,162,641	339	2	no
	T26A8.4	1	exon	-	8,429,493	81	0	no
	W02C12.3	1	exon/intron	+	4,017,054	113	0	no
	Y105C5A.1(flanking)	1	na	+	15,554,689	72	0	no
	Y116A8B.4	1	exon/intron	+	17,223,019	676	1	no
	Y46C8AR.2	0.5	exon	+	3,964,488	566	0	no
	Y73B6BL.14	0.5	exon/intron	-	6,342,282	448	1	no
	Y73B6BL.gc18	1	intron	-	6,354,512	164	0	no
	ZK354.12	1	exon	+	5,316,621	2,294	2	yes
Chromosome V	B0213.11	1	intron	+	3,960,258	112	0	no
	B0213.14	1	intron	-	3,965,262	350	0	no
	C01G10.9	1	exon/intron	+	15,083,215	3,889	12	no
	C12D5.3(flanking)	1	na	-	7,680,479	474	1	no
	C50E3.11	1	intron	-	7,606,251	51	0	no
	F09F3.4	1	exon/intron	-	13,853,597	814	5	no
	F09G2.3	1	intron	+	7,190,190	732	0	yes
	F21C10.12(flanking)	1	na	+	9,101,353	937	2	no
	F29F11.6	1	exon/intron	-	10,685,197	349	0	no
	F37B4.10	1	exon	+	2,876,490	219	1	no
	F47G9.3	0.5	exon/intron	+	11,317,413	4,117	6	no
	F47G9.4	0.5	exon/intron	+	11,317,413	4,117	6	no
	F53F4.8	0.5	exon	-	13,620,160	268	0	no
	F57A10.1	1	exon	+	15,761,874	396	1	yes
	K03D7.11(flanking)	1	na	+	17,522,084	198	0	no
	K04A8.6	1	exon	+	6,536,464	149	0	no
	K11C4.4	1	exon	-	6,901,359	54	0	no
	K11G9.1	1	exon/intron	-	6,680,994	507	0	yes
	M03E7.2	1	exon/intron	-	5,620,508	270	0	no
	R07B7.10	1	exon/intron	+	12,085,882	3,063	5	no
	T04H1.4(flanking)	1	na	-	12,255,428	802	0	no
	T13F3.1	1	exon	-	16,279,988	249	0	no
	Y19D10B.2	1	exon/intron	-	2,301,783	86	0	no
	Y32B12B.2	1	exon/intron	+	16,543,015	363	0	no
	Y50E8A.9	1	exon	-	14,781,068	109	0	no
	ZC404.7	1	intron	-	6,776,482	207	0	no
	ZK1005.1	1	exon	+	1,282,871	82	0	no
	ZK218.12	1	na	+	17,132,534	293	0	yes
	ZK682.4	0.5	exon/intron	+	9,283,235	399	0	no
Chromosome X	C10E2.4	0.5	exon/intron	+	16,750,759	1,455	3	no
	C10E2.5	0.5	exon/intron	+	16,750,759	1,455	3	no
	C14F5.4	1	exon	+	7,958,159	48	0	no
	C36E6.3	1	exon	+	17,459,444	65	0	no
	C42D8.5	1	exon/intron	+	5,094,087	382	0	no
	C44E12.3	1	exon/intron	+	7,603,313	309	0	no
	C53B7.7	0.5	exon/intron	+	6,860,062	3,147	4	no
	F13C5.6(flanking)	1	na	+	622,107	2,192	6	no
	F22A3.2	1	exon	+	6,512,518	26	0	no
	F40F4.8	1	na	+	3,246,609	341	0	no
	F41E7.1	1	exon/intron	+	10,277,835	4,388	12	no
	F52D10.1	1	exon	+	11,585,860	33	0	no
	M03A8.2	0.5	exon	+	6,808,380	766	0	no
	R07A4.1	1	exon/intron	-	10,741,610	319	0	no
	R12H7.1	1	intron	-	13,205,595	605	0	no
	R12H7.4	1	exon/intron	-	13,218,187	678	0	no
	T01B10.5	1	exon/intron	-	8,497,637	174	1	no
	T25B6.7	1	exon/intron	+	9,030,711	341	0	no
	Y7A5A.8	1	intron	-	15,823,834	456	0	no
	ZK1193.1	1	exon	-	421,748	64	0	no
	ZK899.6(flanking)	0.5	na	+	9,465,019	2,042	0	no
mito	MTCE.23	1	exon	-	5,756	258	0	no
	MTCE.25	0.5	na	-	7,439	308	1	no
	MTCE.26	1	exon	-	7,896	48	0	no

Table S4

Table S4. PD3994 SAGE Hits

	gene	SW032	SWN22	SWEG1	SWEM1	SW031	SW030	SW028	SW023	SW033	SW034	SW035	SW037	SW038	SW039	SW040
Chromosome I	C32F10.6(flanking)	7	15	0	0	0	1	0	1	1	1	3	3	0	1	0
	F27C1.11	0	0	0	0	0	0	2	1	0	0	1	3	1	1	0
	F32H2.5	221	63	4	10	15	113	30	16	20	48	86	58	99	4	12
	F47B3.8	1	0	0	0	0	1	2	2	1	3	3	3	4	1	0
	F56C11.1	0	1	0	0	1	0	0	0	1	1	2	0	1	0	0
	K02B12.3	17	9	2	3	7	7	6	1	15	8	10	7	3	3	4
	R06C7.2	1	0	0	0	0	0	0	0	0	2	0	2	0	0	3
	R13H8.1	5	3	1	2	1	0	4	6	2	8	11	5	6	0	0
	T21G5.2	1	5	0	1	0	1	0	0	0	0	0	0	1	0	0
	T26E3.2	0	9	5	4	30	12	5	1	18	17	5	10	11	5	0
	W02A11.3	15	8	3	3	11	14	45	10	4	7	13	7	9	6	3
	Y105E8A.24	3	0	1	0	2	0	6	6	0	5	18	11	13	1	0
	Y106G6D.7(flanking)	1	2	1	0	3	2	2	4	4	2	12	6	6	2	3
	Y18D10A.1	5	0	0	1	0	0	1	4	2	0	0	1	10	2	1
	Y23H5A.3	2	9	0	0	3	1	1	1	1	2	2	1	1	5	13
	Y95B8A.12	0	0	0	0	0	0	5	0	0	0	0	2	0	0	0
	ZC434.3	0	2	0	0	0	0	0	1	0	1	0	1	0	0	0
	ZC434.5	2	1	8	1	3	5	4	1	14	2	2	3	3	2	12
	avg. weighted score:	17.4	7.7	1.3	1.5	4.5	9.5	6.8	3.1	4.5	6.5	9.7	7.2	10.0	1.9	2.6
Chromosome II	B0491.5	47	15	16	12	21	31	11	2	41	16	7	12	7	0	12
	C09H10.3	12	7	4	3	13	18	12	2	29	15	15	11	11	67	19
	C14A4.1	37	14	19	8	31	50	15	4	51	18	16	6	22	22	17
	C15F1.2	1	0	0	0	0	0	0	0	0	0	0	1	0	0	0
	C29H12.1	3	4	0	0	1	0	1	0	0	1	0	3	0	1	4
	C47D12.2	14	14	0	0	5	10	0	2	4	7	8	5	2	0	2
	E04F6.5	3	15	8	5	3	13	10	5	12	11	10	10	4	1	2
	F08B1.1	18	17	8	1	4	15	21	1	11	2	3	5	59	0	2
	F19H8.4	3	0	0	0	0	0	0	0	0	0	3	4	0	1	0
	F28C6.10	1	0	0	0	0	1	0	0	0	0	0	0	0	0	0
	F28C6.8	5	6	5	0	2	1	0	0	6	2	1	0	2	0	1
	F46C5.3(flanking)	0	0	0	0	0	0	0	0	0	0	0	0	2	0	0
	F53A10.2(flanking)	3	5	0	0	5	5	3	0	1	9	4	4	3	6	2
	F58G1.2	3	2	3	0	2	4	2	0	7	0	0	0	6	1	1
	H43E16.1	0	0	0	0	0	0	0	0	0	1	1	1	1	0	0
	R03D7.7	5	1	0	0	0	0	0	0	0	1	0	1	0	0	0
	T08E11.4	1	5	1	0	1	0	0	2	1	3	0	1	3	1	0
	T16A1.7	0	0	3	0	0	0	0	0	1	0	1	0	0	0	0
	T22C8.7	0	1	0	0	0	1	0	1	0	1	0	1	0	0	0
	W02B12.1	1	2	0	0	1	0	0	0	0	0	0	0	1	0	0
	Y48C3A.12	3	1	0	0	1	2	0	2	0	1	1	3	1	7	2
	Y6D1A.1	20	14	1	1	3	1	0	0	1	8	1	4	1	0	3
	ZK938.1	0	0	1	0	0	0	0	0	0	0	0	0	0	0	0
	avg. weighted score:	8.381	5.7	3.2	1.4	4.4	7.2	3.6	1.0	7.7	4.5	3.4	3.4	5.9	5.1	3.2
Chromosome III	C05D10.4	4	12.0	2.0	1.0	3.0	5.0	7.0	0.0	8.0	10.0	9.0	8.0	15.0	9.0	0.0
	C13G5.1	29	9	3	0	3	8	0	1	3	6	4	1	2	2	0
	C14B9.4	42	11	8	5	7	7	22	7	5	18	7	20	11	45	27
	C34E10.8	1	4	1	1	1	1	0	2	1	1	0	1	2	0	0
	C48D5.2	2	7	4	6	8	7	19	6	14	19	11	21	12	16	0
	F44B9.7	0	0	0	0	1	0	0	1	1	1	0	0	0	1	0
	K02F3.6	2	2	1	0	2	4	3	0	2	2	5	4	3	0	0
	K10D2.1	22	8	0	0	1	2	2	1	3	3	7	3	4	5	6
	K11H3.4	1	0	0	0	0	0	0	0	0	1	0	1	3	1	2
	R74.1	17	21	6	3	4	13	14	4	16	5	11	7	13	2	10
	T12D8.1	43	21	4	5	13	14	25	4	7	13	13	17	23	25	3
	T23G5.1	56	26	1	1	4	4	1	5	5	6	3	14	5	1	39
	T26A5.9	45	76	8	49	111	47	2	0	88	4	12	5	2	13	87
	W05G11.6	18	69	1	20	55	28	2	0	11	18	4	21	38	0	0
	Y39E4B.6	16	9	3	4	10	8	3	1	11	13	5	13	4	16	0
	Y48A6C.5	4	0	0	1	0	2	0	2	2	0	8	1	0	0	0
	Y56A3A.5(flanking)	0	0	0	0	0	0	0	0	0	0	0	0	1	0	0
	Y71H2AM.19	0	0	0	0	0	2	1	0	1	1	1	2	1	11	0
	avg. weighted score:	18.5	15.4	2.2	5.9	13.7	8.6	5.3	1.7	11.1	7.0	5.5	7.9	7.1	9.1	11.1

purified oocytes (SW032)-- N2 embryos (SWN22)-- gut cells (SWEG1)-- muscle cells (SWEM1)-- muscle cells (SW031)-- hypodermal cells (SW030)-- pan-neural cells (SW028)-- ciliated neuron (SW023)-- pharynx cells (SW033)-- AFD neuron (SW034)-- pharyngeal marginal (SW035)-- ACER neuron (SW037)-- punc-4::GFP cells (SW038)-- pharyngeal gland cells (SW039)-- dissected gonad (SW040)--

Tables S4-S5. DAM targets are not tissue-specific. For each gene, we assigned a score ("weight") based on its proximity to the nearest cloned *Dpn I* fragment (see "gene score" column in Tables S2 and S3). The weighted "hit frequency" for each gene was then determined by multiplying the gene score by the number of DAM hits for that gene.We then determined the total average weighted hit score for each tissue, by dividing the sum of all weighted hits (for that tissue) by total number of hits (for that tissue). Below the weighted average for each tissue is the average SAGE score (from the SAGE dataset) for the same tissue. In an experiment performed in tandem, we were not able to obtain *Dpn I* fragments from N2 animals (data not shown). And although we were able to capture spurious fragments (i.e. no confirming GATC at the ends) such as vector and random genomic fragments from wildtype animals, it was at a rate at least 10-fold lower than from methylated genomes.

Table S4 (continued)

Table S4 (*cont.*) PD3994 SAGE Hits

	gene	SW032	SWN22	SWEG1	SWEM1	SW031	SW030	SW028	SW023	SW033	SW034	SW035	SW037	SW038	SW039	SW040
**Chromosome IV**	B0513.5	1	1	0	0	1	1	2	2	0	2	2	1	2	0	0
	C29E6.2	2	0	0	0	1	2	1	3	4	3	4	3	2	1	0
	C47E12.6	0	1	0	0	0	8	1	1	1	1	0	2	0	0	0
	F28D1.1	11	8	3	2	5	9	3	3	4	8	9	12	11	40	6
	F32B6.8(flanking)	8	11	2	1	3	11	9	9	8	8	17	9	7	12	10
	F52C12.2	7	9	3	2	4	6	4	3	18	3	2	5	5	3	10
	H23L24.5	2	0	2	0	0	4	0	1	1	0	0	1	34	0	0
	R05C11.3	1	4	0	1	1	2	1	4	1	2	9	1	12	18	0
	T23F6.4	4	7	1	1	2	5	3	1	3	8	12	13	9	1	0
	W03G1.6	13	7	1	0	1	4	3	6	3	3	2	2	3	0	8
	Y2C2A.1	0	2	0	0	0	0	1	2	0	1	2	8	24	0	0
	Y45F10B.10	1	1	0	0	0	0	3	1	1	1	3	1	3	4	0
	Y45F10D.3	21	11	6	1	6	4	6	1	6	9	13	6	4	53	4
	Y54G2A.12	1	0	0	1	0	0	0	1	0	0	0	0	0	0	2
	Y57G11C.33	7	14	1	0	3	4	4	0	15	6	6	4	5	21	8
	Y73F8A.35	0	1	0	0	0	0	0	1	2	0	2	0	2	21	0
	Y94H6A.1	1	0	0	0	0	0	0	0	0	0	1	2	1	0	0
	avg. weighted score:	5.1	4.8	1.2	0.5	1.7	3.8	2.6	2.4	4.3	3.3	4.9	4.2	7.6	11.0	3.1
**Chromosome V**	C03A7.1	0	0	0	0	0	0	0	0	0	0	0	0	1	0	0
	C05E4.3	0	0	0	1	4	1	2	2	0	0	2	3	0	9	0
	C49G7.7	7	0	12	0	0	17	7	3	8	1	1	0	155	2	0
	F11A3.1	6	8	2	2	4	11	1	0	5	2	1	5	0	0	0
	F14H8.6	1	1	0	0	0	2	0	1	0	1	0	0	29	0	0
	F23B12.6	10	7	1	1	3	8	4	5	5	7	8	6	4	1	8
	F58B4.6	0	2	0	0	1	6	12	2	4	0	4	12	13	0	0
	F59A1.11	0	0	0	0	0	0	0	0	0	1	0	0	1	0	0
	K07C5.8	18	11	1	2	3	6	0	0	7	5	5	5	4	3	7
	M03E7.2	0	12	2	1	2	1	1	1	6	1	3	11	11	0	0
	M04G12.1	0	0	3	0	1	4	1	0	0	2	1	1	2	1	0
	T01D3.1	0	0	2	1	1	2	4	7	1	5	6	3	5	1	0
	T04H1.2	9	3	1	0	3	11	2	2	9	5	3	1	14	7	11
	W02F12.3	0	0	0	0	0	0	0	0	0	0	0	0	0	0	3
	W02F12.4	3	8	1	2	1	0	3	3	4	1	0	4	7	1	1
	W04D2.3	1	1	1	0	3	0	1	1	6	1	3	1	3	0	2
	W04D2.5	13	16	26	12	21	26	7	7	44	24	9	9	8	32	11
	Y45G5AM.1	1	0	0	1	0	0	0	0	0	0	0	1	1	2	0
	ZC487.1	0	1	0	0	1	3	3	1	7	3	8	1	3	0	0
	ZK287.8	10	2	1	1	1	2	0	2	2	5	3	3	1	5	0
	avg. weighted score:	4.2	3.4	2.4	1.0	2.1	4.9	2.3	1.9	4.8	3.1	3.0	3.2	15.3	3.0	2.2
**Chromosome X**	C02H7.1	1	1	0	1	0	0	3	3	1	2	3	7	3	0	0
	C17G1.6	1	0	0	0	0	0	0	0	0	0	0	0	3	0	0
	C18B12.5	4	2	1	0	1	2	0	1	1	2	2	1	1	0	0
	C39B10.1	0	1	0	0	0	0	1	0	0	1	1	0	1	0	0
	C39E6.6	0	0	0	0	0	0	3	1	0	0	0	1	0	0	0
	C56E10.1	2	1	2	0	0	0	1	0	2	4	6	3	6	5	0
	E01H11.1	1	3	0	0	1	0	2	1	1	2	2	4	5	0	0
	F41G4.2	18	24	2	12	25	17	9	7	31	34	19	27	19	2	1
	F53A9.3	0	11	2	0	3	3	0	1	17	3	1	4	1	1	0
	F53A9.4	0	1	0	0	1	2	2	2	4	3	15	3	4	0	0
	F54B11.6	13	17	1	2	14	12	14	5	41	28	26	28	10	4	0
	K02D3.1(flanking)	3	3	0	0	2	1	0	0	0	0	1	1	1	1	0
	K03E6.3(flanking)	0	1	0	0	0	0	1	0	0	0	0	0	1	0	0
	K10B3.1	13	26	0	1	7	26	0	0	11	0	0	1	0	0	0
	M02D8.1(flanking)	0	0	0	3	15	0	0	0	6	1	2	0	1	0	0
	R03E1.2	12	24	3	3	8	12	2	0	9	7	1	3	8	4	2
	T23E7.2	3	3	0	4	5	1	1	1	2	6	6	1	5	2	0
	ZC8.6	0	0	0	0	0	0	1	0	1	1	1	1	0	6	1
	ZK899.8	10	18	3	4	2	6	11	7	14	5	11	10	18	18	2
	avg. weighted score:	4.1	6.6	0.5	1.7	4.6	4.2	2.6	1.5	6.8	4.8	4.6	4.4	4.3	2.2	0.3
	**total weighted avg (cloning):**	**9.5**	**7.0**	**1.9**	**1.9**	**4.9**	**6.4**	**3.8**	**1.9**	**6.5**	**4.8**	**5.1**	**4.9**	**8.4**	**5.2**	**3.6**
	**total avg. (SAGE db):**	**7.5**	**6.7**	**2.7**	**1.8**	**4.8**	**6.0**	**3.8**	**2.6**	**7.3**	**4.3**	**5.4**	**4.1**	**5.7**	**4.3**	**4.9**
	**weighted standard error:**	6.3	1.5	0.1	0.3	1.8	2.1	0.5	0.1	1.7	0.6	1.0	0.6	4.2	1.3	1.1
		purified oocytes (SW032)--	N2 embryos (SWN22)--	gut cells (SWEG1)--	muscle cells (SWEM1)--	muscle cells (SW031)--	hypodermal cells (SW030)--	pan-neural cells (SW028)--	ciliated neuron (SW023)--	pharynx cells (SW033)--	AFD neuron (SW034)--	pharyngeal marginal (SW035)--	ACER neuron (SW037)--	punc-4::GFP cells (SW038)--	pharyngeal gland cells (SW039)--	dissected gonad (SW040)--

Table S5

Table S5. PD5122 SAGE Hits

	gene	SW032	SWN22	SWEG1	SWEM1	SW031	SW030	SW028	SW023	SW033	SW034	SW035	SW037	SW038	SW039	SW040
Chromosome I	C16C2.3	2	10	1	0	3	1	1	0	3	0	5	2	5	0	1
	C30F12.3	0	0	0	0	0	0	0	0	1	0	0	0	0	0	0
	C35E7.6	0	0	0	0	0	0	0	0	0	2	0	0	2	0	0
	C41G7.1	11	15	1	1	6	10	4	9	6	8	24	8	2	0	10
	D2030.8	12	7	0	0	0	0	1	1	2	3	1	3	2	0	5
	F14B4.2(flanking)	7	14	4	2	2	20	6	3	15	6	10	3	21	0	29
	F18C12.2	8	4	2	1	0	0	1	2	9	2	5	2	5	2	1
	F26E4.10	10	3	0	0	1	1	3	0	2	1	3	3	5	1	0
	F30A10.10	34	12	5	2	2	6	5	22	10	10	17	18	10	2	0
	F32B4.4	4	4	2	0	1	3	0	0	5	5	3	0	1	7	1
	F32B4.4	4	4	2	0	1	3	0	0	5	5	3	0	1	7	1
	F33D11.8(flanking)	2	0	0	1	1	0	0	0	4	0	0	0	6	0	0
	H05L14.2	0	0	0	0	0	0	0	1	0	0	2	2	1	0	0
	K02A11.1	18	31	4	4	4	11	7	7	22	8	15	5	16	0	1
	R05D11.3	31	11	5	2	11	8	5	1	10	2	5	1	6	1	42
	R119.5	5	3	2	0	3	2	7	1	4	3	3	2	2	36	0
	T04D3.1(flanking)	3	2	0	0	0	0	0	0	0	1	1	1	0	0	1
	T09B4.6(flanking)	34	49	4	10	8	19	0	0	21	0	0	0	0	0	0
	T12F5.4	4	5	2	2	0	7	10	8	5	13	8	5	21	15	2
	T22H2.6	2	18	13	1	3	3	2	0	6	4	1	2	1	1	0
	T27A3.1	6	8	3	2	6	6	3	2	7	2	1	1	6	0	1
	W06D4.1	21	31	9	0	4	61	7	4	8	9	15	12	9	0	1
	Y26D4A.14	0	0	0	0	0	0	0	0	0	0	0	0	1	0	0
	Y48G8AL.1	9	4	1	0	0	2	1	2	0	5	3	3	5	27	5
	Y48G8AL.10	1	0	0	0	0	0	1	0	0	4	0	0	1	1	0
	Y65B4BL.6(flanking)	0	15	5	2	7	12	3	3	22	6	4	13	5	1	0
	ZK973.6	55	8	4	4	6	13	56	35	12	50	56	50	191	219	1
	avg. weighted score:	10.3	9.7	2.6	1.3	2.5	7.2	4.8	3.4	6.8	5.6	6.6	4.9	12.8	12.5	3.8
Chromosome II	B0457.1	13	29	5	3	5	8	12	1	12	12	7	10	13	2	7
	B0495.5	19	9	1	1	3	2	4	0	3	3	6	4	5	1	6
	C08E3.3	0	0	0	0	0	0	0	0	0	0	1	1	0	0	0
	F02E11.1	0	0	0	0	0	0	0	0	0	0	0	1	1	0	0
	F07A11.6	57	15	3	2	3	12	169	20	7	41	52	43	35	51	0
	F08G2.4	8	11	6	3	11	5	1	0	16	3	4	3	11	1	1
	F35D11.5	3	7	6	2	10	3	4	1	18	3	3	2	6	1	13
	F44F4.1	0	0	0	0	0	0	0	0	0	0	1	1	1	0	0
	F49E12.6(flanking)	28	18	9	3	10	3	7	1	14	10	10	9	1	0	0
	M03A1.3	2	2	0	0	0	2	2	3	1	0	3	1	2	2	0
	T06D8.1	1	1	0	0	0	1	0	0	0	0	0	0	0	0	0
	W02B12.3	9	18	1	1	6	7	13	8	22	16	21	3	8	49	19
	Y48E1B.13	1	0	0	0	0	0	1	0	0	0	0	0	1	0	0
	avg. weighted score:	11.0	8.4	2.2	1.1	3.4	3.2	17.0	2.7	6.8	6.9	8.5	6.1	6.3	8.5	3.6
Chromosome III	B0284.1	0	0	0	0	0	2	0	1	0	0	0	0	0	0	0
	B0412.2	0	0	0	0	0	0	0	6	0	0	1	0	38	0	0
	B0523.5	16	10	4	4	4	3	3	1	7	7	14	6	6	3	3
	B0524.1(flanking)	1	0	0	0	0	0	2	3	0	2	3	1	2	1	0
	D2045.9	5	3	0	1	6	2	2	0	3	0	8	2	1	1	0
	F11F1.7	0	0	0	0	0	0	0	0	0	0	0	1	0	0	0
	K10D2.6	20	12	8	0	1	9	8	3	6	6	11	1	5	0	10
	R107.1	98	7	6	0	4	9	3	0	4	3	2	2	1	4	0
	T12B5.11	5	4	2	0	1	0	3	1	4	1	5	3	5	0	0
	Y111B2A.19	0	1	0	1	0	0	0	0	0	0	0	0	0	0	1
	Y47D3A.6	1	2	1	3	2	1	1	0	4	0	0	1	1	1	0
	Y48A6C.5	4	0	0	1	0	2	0	2	2	0	8	1	0	0	0
	Y48A6C.5	4	0	0	1	0	2	0	2	2	0	8	1	0	0	0
	Y56A3A.1	15	6	2	1	3	9	9	6	4	6	10	11	9	21	14
	Y75B8A.1	0	2	0	0	2	3	0	0	0	0	0	0	1	3	0
	ZC84.6	0	3	0	1	1	1	2	3	3	2	7	1	1	9	0
	ZK643.3	0	0	0	0	0	0	0	1	0	0	0	0	4	0	0
	ZK643.5	19	19	8	5	14	22	23	4	33	15	16	13	31	3	0
	ZK783.4	27	15	4	3	3	7	10	4	8	14	14	14	19	3	0
	avg. weighted score:	11.0	3.8	1.7	1.0	1.9	3.3	2.8	1.9	3.4	2.4	5.3	2.5	5.5	2.6	1.6
		purified oocytes (SW032)--	N2 embryos (SWN22)--	gut cells (SWEG1)--	muscle cells (SWEM1)--	muscle cells (SW031)--	hypodermal cells (SW030)--	pan-neural cells (SW028)--	ciliated neuron (SW023)--	pharynx cells (SW033)--	AFD neuron (SW034)--	pharyngeal marginal (SW035)--	ACER neuron (SW037)--	punc-4::GFP cells (SW038)--	pharyngeal gland cells (SW039)--	dissected gonad (SW040)--

Table S5 (continued)

Table S5 (*cont.*) PD5122 SAGE Hits

Chromosome	gene	SW032	SWN22	SWEG1	SWEM1	SW031	SW030	SW028	SW023	SW033	SW034	SW035	SW037	SW038	SW039	SW040
Chromosome IV	4R79.1	0	3	2	0	1	1	1	1	1	2	6	4	9	0	0
	B0350.2	43	25	16	36	82	34	93	39	46	78	79	90	139	105	1
	C01C7.1	2	9	1	11	12	3	12	6	2	6	12	4	13	0	1
	C33H5.9	5	5	1	3	4	6	0	1	4	1	8	1	2	1	2
	C46G7.2	4	12	1	11	55	6	0	0	8	3	0	2	0	0	1
	F08B4.1	1	7	1	1	1	4	3	5	2	1	1	2	6	0	0
	F29B9.8	1	3	0	6	4	2	0	2	0	1	1	1	0	0	1
	F29B9.8	1	3	0	6	4	2	0	2	0	1	1	1	0	0	1
	F32B6.8	8	11	2	1	3	11	9	9	8	8	17	9	7	12	10
	F55A8.2	21	19	0	3	6	18	16	10	10	9	15	9	14	5	19
	R13A1.4	0	1	0	0	0	1	0	0	0	0	1	2	3	2	0
	T01G1.1	14	4	4	3	5	7	10	13	15	12	25	14	36	3	1
	T14G10.1	19	32	2	1	6	17	12	7	26	3	7	13	10	4	11
	T26A8.4	16	8	3	4	4	17	8	8	16	3	5	9	23	0	4
	W02C12.3	7	4	2	5	5	5	2	5	3	2	2	5	9	12	1
	Y105C5A.1(flanking)	4	5	0	0	1	0	3	0	1	2	3	4	1	1	0
	Y73B6BL.14	0	0	0	0	0	0	0	0	0	0	1	0	0	0	2
	avg. weighted score:	8.9	9.1	2.1	5.7	12.0	8.0	10.3	6.5	8.6	8.0	10.9	10.3	16.8	8.7	3.1
Chromosome V	B0213.11	0	0	0	0	0	0	0	1	0	0	0	0	0	0	0
	B0213.14	1	0	0	0	0	0	0	0	0	0	0	0	0	0	0
	C01G10.9	2	0	1	0	0	2	1	1	2	5	2	0	1	9	0
	C12D5.3(flanking)	0	0	0	0	0	0	0	0	0	0	0	1	4	1	0
	C50E3.11	0	0	0	0	0	0	1	0	0	0	0	0	0	0	0
	F09G2.3	1	0	0	0	0	0	0	0	0	0	0	0	0	0	0
	F21C10.12(flanking)	0	0	0	0	0	0	0	0	2	0	1	0	0	0	0
	F29F11.6	9	6	3	1	3	13	1	2	7	4	8	0	5	2	13
	F37B4.10	4	7	3	1	2	6	11	13	13	0	60	7	6	2	2
	F47G9.3	0	0	0	0	0	0	1	0	2	1	1	1	1	1	0
	F47G9.4	0	0	0	1	1	0	0	0	0	1	4	2	1	0	0
	F53F4.8	2	0	2	1	0	0	1	1	0	0	0	0	6	0	0
	K04A8.6	6	39	11	6	11	29	9	4	26	20	6	17	19	2	0
	K11C4.4	235	10	8	2	4	10	3	1	2	7	5	5	1	3	0
	K11G9.1	0	0	0	0	1	1	0	0	0	0	0	1	5	1	0
	M03E7.2	0	12	2	1	2	1	1	1	6	1	3	11	11	0	0
	R07B7.10	2	1	1	0	0	2	1	2	2	1	5	0	0	10	1
	T04H1.4(flanking)	55	13	0	2	3	3	8	1	5	6	12	6	13	6	0
	T13F3.1	10	0	0	0	0	0	0	0	0	0	0	0	0	0	0
	Y19D10B.2	0	1	0	0	0	0	0	0	0	0	0	0	0	0	0
	Y32B12B.2	5	6	1	1	1	4	2	0	1	2	2	1	3	17	5
	ZK1005.1	8	5	8	0	0	1	1	1	0	1	1	2	12	1	0
	ZK682.4	0	0	1	0	0	0	1	5	4	1	8	6	0	0	0
	avg. weighted score:	16.1	4.8	1.9	0.7	1.3	3.4	1.9	1.4	3.3	2.3	5.3	2.6	4.0	2.6	1.0
Chromosome X	C10E2.4	1	0	0	1	0	0	0	0	2	0	0	0	1	0	0
	C14F5.4	1	5	1	1	3	2	1	1	13	5	3	4	2	0	0
	C36E6.3	24	74	5	39	165	18	10	19	35	21	13	27	98	2	2
	C42D8.5	4	11	1	2	1	32	3	2	6	4	5	11	4	0	0
	C44E12.3	0	2	0	0	0	1	12	1	4	2	2	1	2	0	1
	F13C5.6(flanking)	5	18	2	11	18	2	1	9	1	15	17	8	21	0	0
	F22A3.2	2	23	6	41	99	15	2	1	10	3	1	2	0	0	0
	F40F4.8	0	0	1	0	0	0	0	0	0	0	0	0	2	0	0
	F41E7.1	0	0	0	0	0	0	0	1	0	2	5	1	2	0	0
	F52D10.1	1	0	0	0	0	0	0	0	0	0	0	0	0	0	1
	M03A8.2	6	9	2	3	1	7	11	14	10	24	38	19	13	39	0
	R07A4.1	0	0	0	0	1	0	1	0	2	0	1	0	1	1	0
	R12H7.1	1	7	3	3	10	2	12	4	7	4	4	4	16	1	0
	R12H7.4	0	1	0	0	1	0	0	0	0	1	0	0	1	0	0
	T01B10.5	0	0	2	0	1	1	1	1	2	1	2	0	2	0	0
	T25B6.7	1	1	0	0	0	0	1	0	0	1	0	0	0	0	0
	ZK1193.1	2	0	0	0	0	0	0	0	0	0	0	0	0	0	11
	ZK899.6(flanking)	12	8	0	1	3	1	0	0	1	1	0	0	0	0	0
	avg. weighted score:	3.1	9.1	1.3	6.0	18.2	4.7	3.0	2.8	5.2	4.3	4.4	4.1	9.6	1.4	0.9
	**total weighted avg (cloning):**	**10.3**	**7.5**	**2.0**	**2.5**	**6.1**	**5.1**	**5.9**	**3.1**	**5.6**	**4.7**	**6.7**	**4.9**	**9.3**	**6.3**	**2.3**
	**total avg. (SAGE db):**	**7.5**	**6.7**	**2.7**	**1.8**	**4.8**	**6.0**	**3.8**	**2.6**	**7.3**	**4.3**	**5.4**	**4.1**	**5.7**	**4.3**	**4.9**
	**weighted standard error:**	6.4	1.2	0.1	0.4	3.9	0.7	3.4	0.4	0.6	1.0	1.4	1.2	5.7	5.5	0.3

purified oocytes (SW032)-
N2 embryos (SWN22)-
gut cells (SWEG1)-
muscle cells (SWEM1)-
muscle cells (SW031)-
hypodermal cells (SW030)-
pan-neural cells (SW028)-
ciliated neuron (SW023)-
pharynx cells (SW033)-
AFD neuron (SW034)-
pharyngeal marginal (SW035)-
ACER neuron (SW037)-
punc-4::GFP cells (SW038)-
pharyngeal gland cells (SW039)-
dissected gonad (SW040)-
